# A pathogen-derived effector modulates host glucose metabolism by arginine GlcNAcylation of HIF-1α protein

**DOI:** 10.1371/journal.ppat.1007259

**Published:** 2018-08-20

**Authors:** Chenxi Xu, Xing Liu, Huangyuan Zha, Sijia Fan, Dawei Zhang, Shan Li, Wuhan Xiao

**Affiliations:** 1 State Key Laboratory of Freshwater Ecology and Biotechnology, Institute of Hydrobiology, Chinese Academy of Sciences, Wuhan, P. R. China; 2 University of Chinese Academy of Sciences, Beijing, P. R. China; 3 Institute of Infection and Immunity, Taihe Hospital, Hubei University of Medicine, Shiyan, P. R. China; 4 Biomedical Center, College of Life Science and Technology, Huazhong Agricultural University, Wuhan, P. R. China; 5 The Key laboratory of Aquaculture Disease Control, Ministry of Agriculture, Wuhan, P. R. China; McMaster University, CANADA

## Abstract

The essential role of pathogens in host metabolism is widely recognized, yet the mechanisms by which they affect host physiology remain to be fully defined. Here, we found that NleB, an enteropathogenic *Escherichia coli* (EPEC) type III secretion system effector known to possess N-acetylglucosamine (GlcNAc) transferase activity, GlcNAcylates HIF-1α, a master regulator of cellular O_2_ homeostasis. We determined that NleB-mediated GlcNAcylation at a conserved arginine 18 (Arg18) at the N-terminus of HIF-1α enhanced HIF-1α transcriptional activity, thereby inducing HIF-1α downstream gene expression to alter host glucose metabolism. The arginine transferase activity of NleB was required for its enhancement of HIF-1α transactivity and the subsequent effect on glucose metabolism in a mouse model of EPEC infection. In addition, HIF-1α acted as a mediator to transact NleB-mediated induction of glucose metabolism-associated gene expression under hypoxia. Thus, our results further show a causal link between pathogen infection and host glucose metabolism, and we propose a new mechanism by which this occurs.

## Author summary

Accumulating evidence shows that pathogens can affect host metabolism, resulting in human diseases such as obesity and type 2 diabetes. However, how pathogens influence their hosts is still not clear, and this results in a lack of effective and specific clinical treatments. Further investigations into the causes of pathogen disturbance of host metabolism are urgently needed. In this study, we show that a protein molecule, NleB, secreted by enteropathogenic bacteria (EPEC) can get into host cells and modify the function of a master regulator of cellular O_2_ homeostasis, HIF-1α, thereby altering host glucose metabolism. We show that HIF-1α acts as a mediator to transact NleB-mediated induction of glucose metabolism-associated gene expression under hypoxia. Our results reveal a causal link between pathogen infection and host glucose metabolism, which may provide a new explanation for the causes of human diseases related to metabolic disturbance.

## Introduction

Over the past decade, it has become clear that pathogens play an important role in host metabolism, but the mechanisms by which they affect host metabolism remain poorly defined [1–10]. Although pathogenic organisms likely rely on effectors to communicate with their host, the identity and function of pathogen-encoded effectors remain largely unknown [2,8,9]. Identifying pathogen effectors that affect host metabolism and defining the pathways that effectors use to enact their changes to host metabolism can help us better understand the relationship between pathogen infection and host physiology, as well as provide insights into the mechanisms underlying human diseases related to metabolic disturbance, such as obesity and type 2 diabetes (T2DM).

Human pathogenic *Escherichia coli*, enteropathogenic *E*. *coli* (EPEC), are attaching/effacing (A/E) pathogens that are the causative agent of infantile diarrhea. EPEC, enterohemorrhagic *E*.*coli* (EHEC), and the mouse pathogen *Citrobacter rodentium* all use a type III secretion system (T3SS) to transfer effectors (virulence proteins) into host intestinal epithelial cells to modulate various cell functions [11] [12]. The EPEC T3SS effector NleB, a protein known to inhibit host nuclear factor kappa-light-chain-enhancer of activated B cells (NF-kB) signaling [12], has recently been found to possess N-acetyglucosamine (GlcNAc) transferase activity that specifically modifies conserved arginine residues in death domain-containing host proteins, including TRADD, FADD, RIPK1, and TNFR1 [12–15]. Additionally, GAPDH is another target that becomes glycosylated by NleB at Arg197 and Arg200 [16], though this is debatable [13]. Due to this unprecedented function, NleB and its homologous effectors may modify additional host targets beyond those already identified [16,17]. Defining additional targets and demonstrating the consequences and underlying mechanisms of NleB-modified host targets will not only help us understand the correlation between pathogen and host physiology, but also provide an opportunity to discover therapeutic strategies to combat human diseases related to these pathogens.

Oxygen (O_2_) is a key factor for balancing demand against substrate availability in the glucose metabolic response, affecting both the rate and pathway of substrate utilization for energy production [18]. Based on O_2_ availability, HIF-1α, which is a master regulator of cellular O_2_ homeostasis, regulates glycolytic and oxidative glucose metabolism gene expression in the glucose metabolism pathway [18–20] [21–25]. Other host factors are already known to modulate HIF-1α function and thus also influence cellular glucose homeostasis [26,27], but other HIF-1α modulators may exist as well [20].

The intestines maintain low O_2_ conditions due to counter-current blood flow [28]. Multiple lines of evidence indicate that pathogens greatly influence host glucose metabolism, but the major underlying mechanisms remain largely unknown [10,20]. We found here that the T3SS effector NleB from EPEC modifies arginine residues in host HIF-1α through GlcNAcylation, thereby enhancing HIF-1 signaling. Moreover, the arginine GlcNAc transferase activity of NleB is required for enhancing host glucose metabolism in *C*. *rodentium*-infected mice. These results provide a causal, mechanistic link to explain how pathogens modulate host glucose metabolism.

## Results

### NleB GlcNAcylates HIF-1α protein at arginine sites

**Fig 1:**
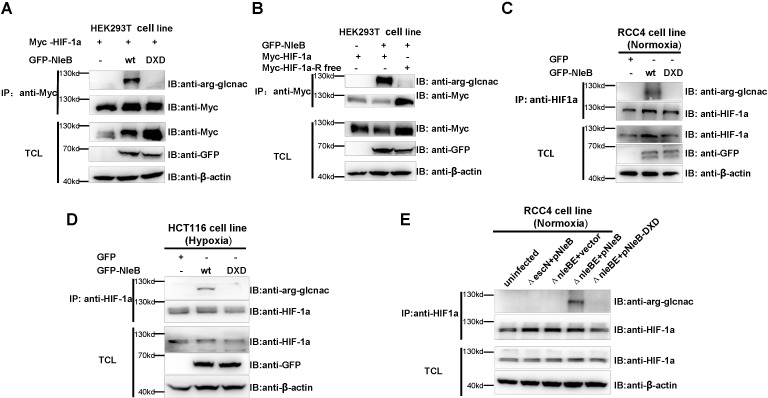
NleB GlcNAcylates HIF-1α protein at arginine residues. (A) Effects of NleB transfection on arginine GlcNAcylation of HIF-1α in HEK293T cells. IP, immunoprecipitation; TCL, total cell lysates; GFP-NleB, GFP-tagged wild-type NleB; GFP-NleB-DXD, GFP-tagged NleB Asp221Ala/Asp223Ala double mutant. (B) Effects of NleB transfection on GlcNAcylation of HIF-1α arginine mutant in HEK293T cells. HIF-1α-R-free, all 35 arginine residues in human HIF-1α were mutated to lysine residues. (C) Effects of NleB transfection on arginine GlcNAcylation of endogenous HIF-1α in RCC4 cells. (D) Effects of NleB transfection on arginine GlcNAcylation of endogenous HIF-1α in HCT116 cells under hypoxia. (E) Endogenous HIF-1α in RCC4 cells was GlcNAcylated by T3SS-delivered NleB. RCC4 cells were uninfected or infected with a mutant EPEC strain lacking *escN* (indicated as Δ*escN*) but complemented with a plasmid expressing WT NleB (Δ*escN*+pNleB); or infected with mutant EPEC strains lacking both *nleE* and *nleB* (strain SC309, indicated as Δ*nleBE*) but complemented with an empty plasmid (Δ*nleBE*+vector), a plasmid expressing wild-type NleB (Δ*nleBE*+pNleB), or a plasmid expressing the GlcNAc transferase-deficient D221A/D223A mutant (Δ*nleBE*+pNleB-DXD). Cell lysates were subjected to anti-HIF-1α IP and detected by an anti-arginine (GlcNAc) antibody. IP, immunoprecipitation; TCL, total cell lysates; IB, immunoblotting.

While studying HIF-1α function, we noted that some arginine-to-lysine (R/K) mutations affected HIF-1α activity. However, none of the arginine-modifying factors we screened (such as arginine methyltransferases [PRMTs]) accounted for the effect displayed in HIF-1α R/K mutants. Two recent studies have shown that the EPEC T3SS effector NleB modifies host proteins through arginine GlcNAcylation [13,14], prompting us to investigate whether bacterial NleB can modify host HIF-1α by arginine GlcNAcylation and thereby affect host glucose metabolism [20] [1,2,18–20]. We first tested whether the wild-type NleB protein could GlcNAcylate HIF-1α by co-expressing GFP-tagged wild-type NleB (GFP-NleB) together with Myc-tagged HIF-1α (Myc-HIF-1α) in HEK293T cells. A clear band was detected by anti-arginine (glcnac) antibody (ab195033, Abcam) after the lysates were co-immunoprecipitated (co-IP) with anti-Myc antibody-conjugated agarose beads [17,29], but this band was not present when Myc-HIF-1α was co-expressed with a GFP-tagged catalytic motif (DXD) mutant NleB (GFP-NleB-DXD, Asp221Ala/Asp223Ala double mutant) (Fig 1A). These observations suggest that NleB might GlcNAcylate HIF-1α at arginine residues [29], and that the arginine GlcNAc transferase activity of NleB is required for this GlcNAcylation.

To confirm that NleB indeed GlcNAcylated HIF-1α at arginine residues, we made an R/K HIF-1α mutant (HIF-1α-R-free, in which all 35 arginine residues were mutated to lysine residues) and examined the ability of NleB to GlcNAcylate it. After co-expression and co-IP, the anti-arginine (glcnac) antibody could not detect any GlcNAcylated residues in HIF-1α-R-free, indicating that NleB could indeed GlcNAcylate arginine(s) in HIF-1α (Fig 1B).

To determine whether NleB could GlcNAcylate endogenous HIF-1α, we took advantage of the RCC4 cell line, an VHL-deficient kidney cancer cell line in which HIF-α (HIF-1α and HIF-2α) is highly expressed under normoxia [30]. When GFP-NleB was overexpressed, an clear band was detected by anti-arginine (glcnac) antibody after the lysates were co-immunoprecipitated with anti-HIF-1α antibody, but this band was not present when GFP-NleB-DXD was overexpressed (Fig 1C). This data suggests that NleB GlcNAcylates endogenous HIF-1α.

To determine whether NleB could GlcNAcylate endogenous HIF-1α under hypoxia, we used the HCT116 cell line, in which endogenous HIF-1α is induced by hypoxia. Similar to what was exhibited in the RCC4 cell line, when GFP-NleB was overexpressed, an clear band was detected by anti-arginine (glcnac) antibody after the lysates were co-immunoprecipitated with anti-HIF-1α antibody, but this band was not present when GFP-NleB-DXD was overexpressed (Fig 1D). This data suggests that NleB GlcNAcylates endogenous HIF-1α under hypoxia.

Subsequently, to determine whether EPEC T3SS-delieved NleB could GlcNAcylate endogenous HIF-1α similar to NleB overexpression, we used the wild-type EPEC strain (E2348/69) and the mutant EPEC strains with different genetic backgrounds after they were complemented with various genes (S1 Table; S1 Fig; Fig 1E). In EPEC, NleB is encoded directly upstream from NleE, another type III effector [12,31]. In addition, it has been reported previously that both NleB and NleE can suppress NF-kB activation [12,31]. To exclude the potential effects of NleE on HIF-1α, we used a mutant EPEC strain with double deletion of *nleE* and *nleB* (Δ*nleBE*) for subsequent assays, similar to that used by Li et al. [13]. HIF-1α GlcNAcylation was detected only in RCC4 cells infected with the wild-type EPEC (E2348/69) (S1 Fig) and in the *nleE-* and *nleB-*deleted mutant EPEC strain complemented with a plasmid expressing wild-type NleB (Δ*nleBE*+pNleB), but not in uninfected cells or in cells infected with the *escN-*deleted EPEC mutant strain (which is defective in its T3SS delivery system and is unable to deliver proteins into host cells) complemented with a plasmid expressing wild-type NleB (Δ*esc*N+pNleB), the control Δ*nleBE*+vector, or Δ*nleBE*+pNleB-DXD (S1 Fig; Fig 1E).

This observation suggests that NleB delivered by bacteria efficiently GlcNAcylates endogenous HIF-1α in host cells, and that the T3SS machinery is required for NleB to GlcNAcylate endogenous HIF-1α.

**Fig 2:**
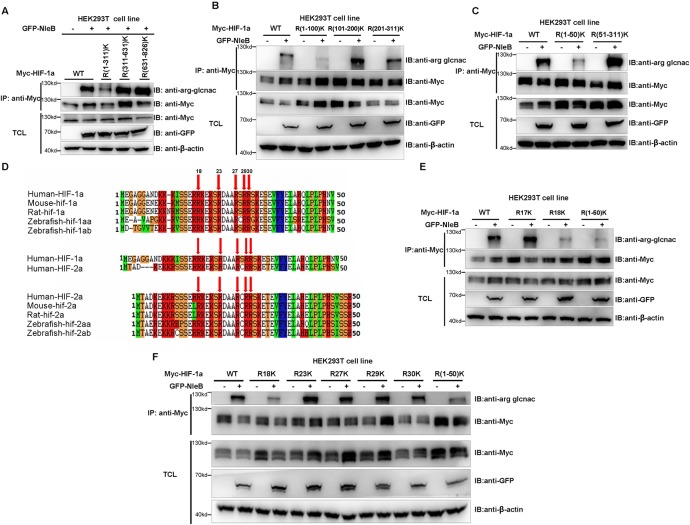
NleB GlcNAcylates HIF-1α protein mainly at arginine 18. (A) Effects of NleB transfection on arginine GlcNAcylation of HIF-1α and its arginine mutants in HEK293T cells. WT, wild-type human HIF-1α; R(1–311)K, 20 arginine residues (R17, R18, R23, R27, R29, R30, R61, R68, R70, R145, R151, R165, R170, R178, R180, R245, R258, R273, R306, and R311) in the N-terminal domain (amino acids 1–311) of human HIF-1α were simultaneously mutated to lysine residues; R(311–631)K, 5 arginine residues (R311, R463, R507, R575, and R631) in the middle domain (amino acids311-631) of human HIF-1α were simultaneously mutated to lysine residues; R(631–826)K, 12 arginine residues (R631, R660, R665, R671, R686, R698, R718, R720, R754, R781, R810, and R820) in the C-terminal domain (amino acids 631–826) of human HIF-1α were simultaneously mutated to lysine residues; IP, immunoprecipitation; TCL, total cell lysates; GFP-NleB, GFP tagged wild-type NleB. (B) Effects of NleB transfection on GlcNAcylation of HIF-1α and its arginine mutants in N-terminal domain subregions in HEK293T cells. WT, wild-type human HIF-1α; R(1–100)K, 8 arginine residues (R17, R18, R23, R27, R29, R30, R61, and R68) in the 1–100 amino acid region of human HIF-1α were simultaneously mutated to lysine residues; R(101–200)K, 6 arginine residues (R145, R151, R165, R170, R178, and R180) in the 101–200 amino acid region of human HIF-1α were simultaneously mutated to lysine residues; R(201–311)K, 5 arginine residues (R245, R258, R273, R306, and R311) in the 201–311 amino acid region of human HIF-1α were simultaneously mutated to lysine residues. (C) Effects of NleB transfection on GlcNAcylation of HIF-1α and its arginine mutants in different N-terminal domain subregions in HEK293T cells. WT, wild-type human HIF-1α; R(1–50)K, 6 arginine residues (R17, R18, R23, R27, R29, and R30) in the 1–50 amino acid region of human HIF-1α were simultaneously mutated to lysine residues; R(51–311)K, 14 arginine residues (R61, R68, R70, R145, R151, R165, R170, R178, R180, R245, R258, R273, R306, and R311) in the 51–311 amino acid region of human HIF-1α were simultaneously mutated to lysine residues. (D) Alignments of the N-terminal region (amino acids 1–50) of HIF-1α and HIF-2α from human, mouse, rat, and zebrafish (a and b). Conserved amino acid residues are marked by red arrows. (E) Effects of NleB transfection on GlcNAcylation of HIF-1α and its arginine mutants for Arg17 and Arg18 in HEK293T cells. WT, wild-type human HIF-1α; R17K and R18K, arginine residues at sites 17 or 18 of human HIF-1α were individually mutated to lysine. (F) Effects of NleB transfection on HIF-1α arginine mutants. R18K, R23K, R27K, R29K, and R30K, arginines of sites 18, 23, 27, 29, and 30 in human HIF-1α were individually mutated to lysine; R(1–50)K, 6 arginine residues (R17, R18, R23, R27, R29, and R30) in the N-terminal domain (amino acids 1–50) of human HIF-1α were simultaneously mutated to lysine residues. TCL, Total cell lysates; IP, immunoprecipitation; IB, immunoblotting.

To identify which arginine residue(s) in HIF-1α were GlcNAcylated by NleB, we conducted a series of fine mapping assays using regional R/K HIF-1α mutants. We first narrowed the region down to residues 1–311 (Fig 2A) and then to 1–50 (Fig 2B and 2C). Of the 5 arginine residues, we found that they are evolutionarily conserved between HIF-1α and HIF-2α (Fig 2D). GlcNAcylation of only the R18K mutant was greatly reduced compared to the other 4 single R/K mutants, including the R17 residue next to R18 in the wild-type HIF-1α (Fig 2E and 2F). In addition, mass spectrometry analysis also suggested that Arg18 (R18) was GlcNAcylated by NleB (S2 Table). These data suggest that NleB GlcNAcylates the HIF-1α protein at multiple arginine sites with Arg18 (R18) as the key site.

### NleB interacts with HIF-1α directly

**Fig 3:**
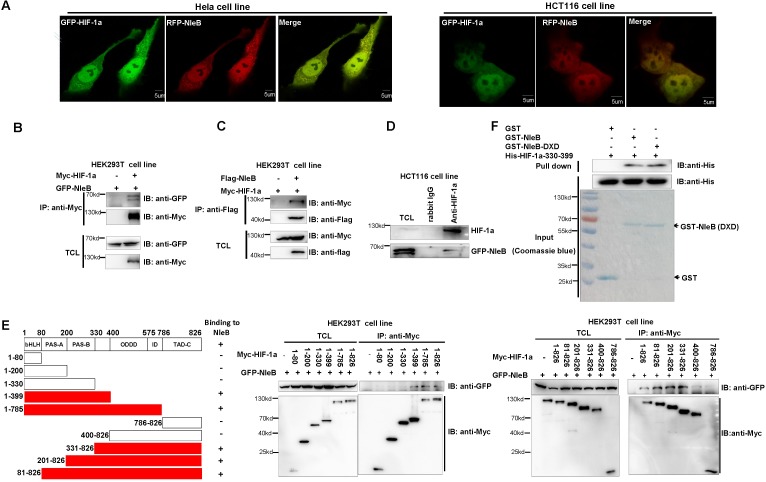
NleB interacts with HIF-1α. (A) HIF-1α co-localized with NleB in HeLa cells and HCT116 cells. HeLa cells and HCT116 cells were transfected with GFP-tagged HIF-1α (GFP-HIF-1α) together with RFP-tagged NleB and then examined by a Zeiss confocal microscope (LSM-710). Pearson’s correlation coefficient (R^2^) for colocalization: HeLa, 0.59; HCT116, 0.76. Scale bar = 5 μM. (B, C) Co-immunoprecipitation assays of NleB interaction with HIF-1α. HEK293T cells transfected with the indicated expression plasmids were subjected to anti-Myc (B) or anti-Flag (C) IP. (D) Co-IP of NleB with endogenous HIF-1α. HCT116 cells transfected with GFP-NleB expression vector were subjected to anti-HIF-1α IP and detected by anti-GFP antibody. (E) Domain mapping for HIF-1α interaction with NleB. NleB interaction with HIF-1α through the middle region of HIF-1α (330–399 aa). The HIF-1α domains that interacted with NleB are marked by red columns (left panel); “-”, no obvious interaction; “+” interaction. (F) Bacterial-expressed GST-tagged NleB (GST-NleB) and GST-tagged NleB-DXD (NleB-DXD) interacted with bacterial-expressed His-tagged HIF-1α (330–399 aa) (His-HIF-1α (330–399 aa)) as revealed by GST pull-down assays. TCL, Total cell lysates; IP, immunoprecipitation; IB, immunoblotting.

To determine whether NleB GlcNAcylates HIF-1α through direct protein–protein interaction, we assessed the interaction between NleB and HIF-1α. Initially, we co-transfected GFP-HIF-1α and RFP-NleB into HeLa cells or HCT116 cells and found that HIF-1α co-localized with NleB (Fig 3A). Subsequently, we co-transfected GFP-NleB and Myc-HIF-1α into HEK293T cells. Myc-HIF-1α pulled-down GFP-NleB when anti-Myc-conjugated agarose beads were used for co-IP (Fig 3B). Confirming this finding, Flag-NleB also reciprocally pulled-down Myc-HIF-1α (Fig 3C), and endogenous HIF-1α could pull-down transfected GFP-NleB in HCT116 cells (Fig 3D).

To further determine which HIF-1α region was essential to bind to NleB, we performed domain mapping and found that the middle region (330–399 amino acids) had a strong ability to bind to NleB (Fig 3E). Interestingly, the R/K mutant, HIF-1α-R-free, still interacted with NleB even though it was not glycosylated by NleB (S2A Fig), and the transferase-deficient NleB-DXD also interacted with wild-type HIF-1α (S2B Fig).

Moreover, glutathione S-transferase (GST)-pull-down assays using GST-tagged NleB and His-tagged HIF-1α (330–399 aa) expressed in *E*. *coli* showed that GST-NleB could pull-down His-HIF-1α-330-399 aa (Fig 3F).

These results suggest that NleB interacts with endogenous HIF-1α directly, and that NleB might GlcNAcylate HIF-1α through direct interaction.

### NleB enhances HIF-1α transcriptional activity

**Fig 4:**
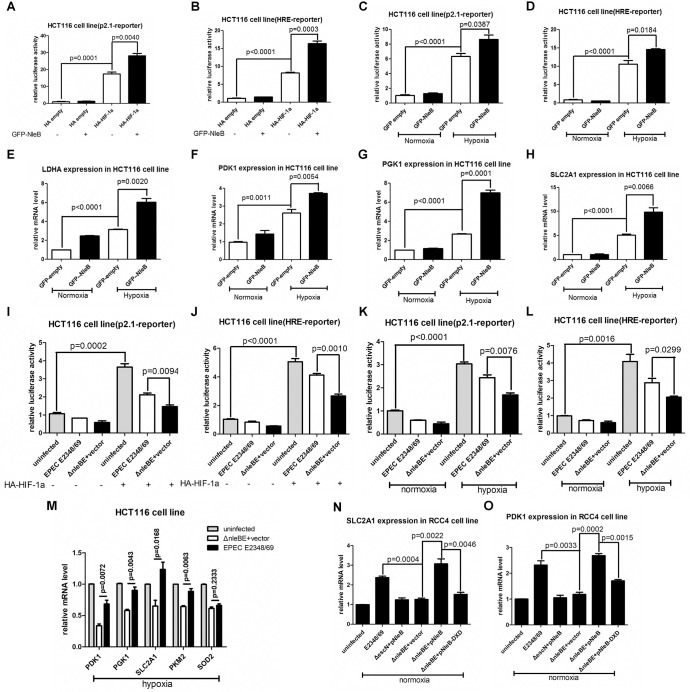
NleB enhances HIF-1α transcriptional activity, and type III-delivered NleB and its GlcNAc transferase activity are required for enhancing HIF-1α transcriptional activity. (A, B) Induction of p2.1-reporter luciferase activity (A) or HRE-reporter luciferase activity (B) by HIF-1α transfection under normoxia was significantly enhanced by NleB overexpression in HCT116 cells (*p =* 0.0040 and *p =* 0.0003, respectively). HRE, hypoxia response element. (C, D) Induction of p2.1-reporter luciferase activity (C) or HRE-reporter luciferase activity (D) under hypoxia was significantly enhanced by NleB overexpression in HCT116 cells (*p =* 0.0387 and *p =* 0.0184, respectively). (E, F, G, H) Induction of *LDHA* (E), *PDK1* (F), *PGK1* (G), or *SLC2A1* (*GLUT1*) (H) expression under hypoxia was significantly enhanced by NleB overexpression in HCT116 cells (*p =* 0.0020, 0.0054, 0.0001, and 0.0066, respectively). (I, J) Induction of p2.1-reporter luciferase activity (I) or HRE-reporter luciferase activity (J) by HIF-1α overexpression under normoxia in HCT116 cells was significantly enhanced by infection with the wild-type EPEC strain (EPEC E2348/69) compared with that by infection with the control EPEC strain lacking both *nleE* and *nleB* (strain SC309) but complemented with an empty plasmid (Δ*nleBE*+vector) (*p =* 0.0094 and *p =* 0.0010, respectively). (K, L) Under hypoxia, the activity of p2.1-luciferase reporter (K) or HRE-luciferase reporter (L) was significantly enhanced in HCT116 by infection with EPEC E2348/69 compared with those by infection with the control Δ*nleBE*+vector (*p =* 0.0076 and *p =* 0.0299, respectively). (M) Under hypoxia, expressions of *PDK1*, *PGK1*, *SLC2A1*, and *PKM2* were significantly enhanced in HCT116 cells by infection with EPEC E2348/69 compared with those by infection with the control Δ*nleBE*+vector, but expression of *SOD2* was not changed. (N, O) Under normoxia, in RCC4 cells, infection with EPEC E2348/69 or the mutant EPEC strain lacking both *nleE* and *nleB* (strain SC309, indicated as Δ*nleBE*) complemented with a plasmid expressing wild-type NleB (Δ*nleBE*+pNleB) enhanced the expression of *SLC2A1* (N) or *PDK1* (O) significantly compared with cells infected with the mutant EPEC strain lacking *escN* (indicated as Δ*escN*) complemented with a plasmid expressing WT NleB (Δ*escN*+pNleB), the control Δ*nleBE*+vector, or the mutant EPEC strain Δ*nleBE*+pNleB-DXD. Data are presented as means + SEM of three independent experiments performed in triplicate.

To determine the biological consequences of NleB-mediated GlcNAcylation of HIF-1α, we examined whether NleB affected HIF-1α transcriptional activity. Initially, we took advantage of luciferase reporter assays using well-defined luciferase reporters to monitor HIF-1α activity [32–37]. NleB expression enhanced p2.1-luciferase reporter and hypoxia response element (HRE)-reporter activity activated by HIF-1α overexpression in HCT116 cells under normoxia [34,35] (Fig 4A and 4B). Under hypoxia, NleB expression significantly enhanced p2.1-luciferase reporter and HRE-reporter activity (Fig 4C and 4D). The expressions of transfected HA-HIF-1α and GFP-NleB were confirmed by western blot assays (S3A, S3B, S3C and S3D Fig).

Because we were particularly interested in whether and how the pathogen correlated with host glucose metabolism, we next selectively examined whether NleB affected glucose metabolism-associated genes known to be downstream of HIF-1α [19,20,25,38–42], including *LDHA*, *PDK1*, *PGK1*, *SLC2A1* (*GLUT1*), *PKM2*, and *HK1* [20]. Consistent with the above reporter assays, NleB expression caused an increase in the mRNA levels of these genes in HCT116 cells under hypoxia as revealed by semi-quantitative RT-PCR assays (Fig 4E, 4F, 4G and 4H). The expressions of transfected GFP-NleB were confirmed by western blot assays (S3E Fig).

Interestingly, NleE also enhanced HIF-1α transcriptional activity as observed by promoter assays in both HCT116 cells and HeLa cells (S4 Fig), further reinforcing the usefulness of the *nleE-* and *nleB-*deleted mutant EPEC strain for analyzing NleB function in this study.

Next, we examined whether EPEC-delivered NleB can enhance HIF-1α transcriptional activity. When HIF-1α was overexpressed in HCT116 cells under normoxia, the wild-type EPEC E2348/69 strain enhanced p2.1-luciferase reporter and hypoxia response element (HRE)-reporter activity compared with the *nleE-* and *nleB-*deleted mutant EPEC strain complemented with an empty control vector (Δ*nleBE*+vector) (Fig 4I and 4J). Similarly, under hypoxia, infection with the wild-type EPEC E2348/69 strain enhanced p2.1-luciferase reporter and hypoxia response element (HRE)-reporter activity compared with infection with the control EPEC strain Δ*nleBE*+vector (Fig 4K and 4L). Of note, the promoter activity in cells without infection was higher than that in infected cells (Fig 4I, 4J, 4K and 4L). This phenomenon was probably due to the unhealthy status of the infected cells, which were not suitable for efficient expression of transfected vectors.

Additionally, we found that NleB also enhanced the promoter activity of BNIP3, which is a HIF-α downstream target responsible for hypoxia-induced cell death [43–45] [46] (S5 Fig), indicating that NleB might impact hypoxia-induced cell death by modulating HIF-α [47].

Consistently, under hypoxia, the infection of the wild-type EPEC E2348/69 strain induced the expressions of *PDK1*, *PGK1*, *SLC2A1* (*GLUT1*), and *PKM2*, four well-defined HIF-1α targets, but did not induce the expression of *SOD2*, a well-defined HIF-2α target (Fig 4M).

To further confirm this induction, we examined the expressions of *SLC2A1* (*GLUT1*) and *PDK1* in RCC4 cells under normoxia. As shown in Fig 4N and 4O, only infection with the wild-type EPEC E2348/69 or the mutant strain Δ*nleBE*+pNleB resulted in significant induction of *SLC2A1* (*GLUT1*) and *PDK1*, but in cells that were infected with the mutant strain Δ*esc*N+pNleB, the control strain Δ*nleBE*+vector, or the mutant strain Δ*nleBE*+pNleB-DXD, there was no significant effect on the induction of *SLC2A1* (*GLUT1*) and *PDK1* (Fig 4N and 4O).

These results suggest that NleB enhances HIF-1α transcriptional activity, that NleB delivered by bacteria efficiently enhances HIF-1α transcriptional activity, and that the T3SS machinery is required for NleB to enhance HIF-1α transcriptional activity.

### The enhancement of HIF-1α transcriptional activity by NleB is dependent on HIF-1α

**Fig 5:**
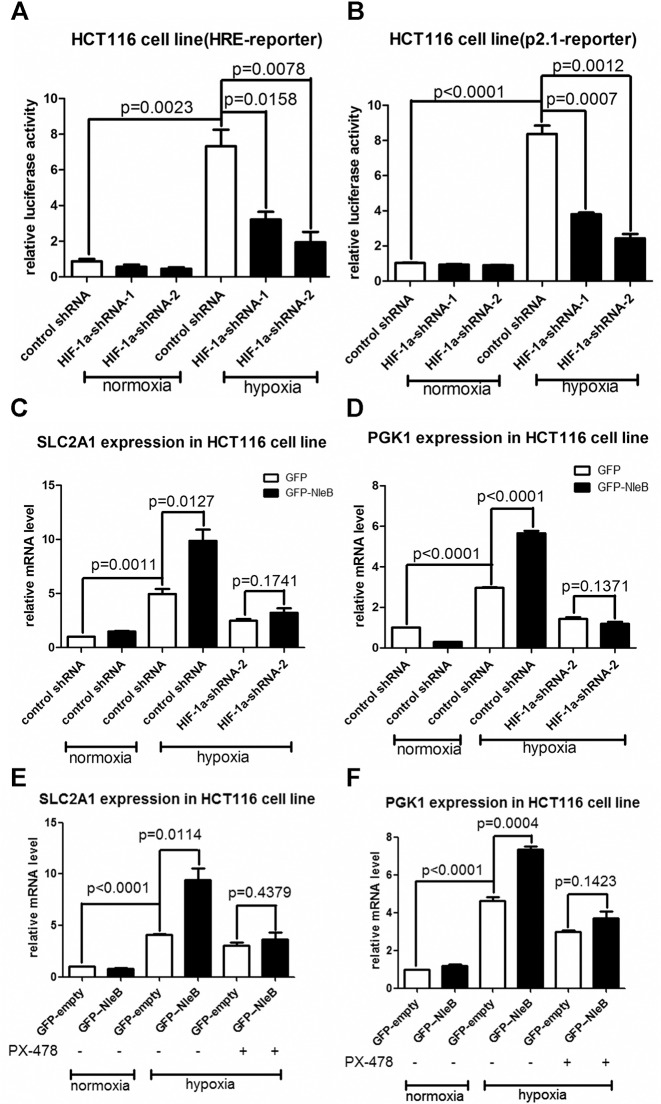
NleB-enhanced hypoxia-inducible gene expression in HCT116 cells is dependent on HIF-1α. (A, B) Knockdown of HIF-1α by HIF-1α-shRNA-1 or HIF-1α-shRNA-2 in HCT116 cells resulted in a reduction of activity of HRE-luciferase reporter (A) or p2.1-luciferase reporter (B) under hypoxia. (C, D) When HIF-1α was knocked-down by HIF-1α-shRNA-2 in HCT116 cells, the enhancement of *SLC2A1* (*GLUT1*) (C) or *PGK1* (D) expression by overexpression of GFP-NleB under hypoxia was diminished (*p =* 0.1741 and *p =* 0.1371, respectively). (E, F) When the HIF-1α inhibitor PX-478 was added into HCT116 cells, the enhancement of *SLC2A1* (*GLUT1*) (E) or *PGK1* (F) expression by overexpression of GFP-NleB under hypoxia was diminished (*p =* 0.4379 and *p =* 0.1423, respectively). Data are presented as means + SEM of three independent experiments performed in triplicate.

To determine whether the effect of NleB on hypoxia signaling is mediated by HIF-1α, we initially knocked-down HIF-1α by transient transfection of HIF-1α shRNA in HCT116 cells (S6 Fig). As shown in Fig 5A and 5B, when HIF-1α shRNAs were co-transfected, the activities of the p2.1-luciferase reporter and hypoxia response element (HRE) reporter induced under hypoxia were reduced significantly (Fig 5A and 5B), indicating the efficiency of HIF-1α shRNAs (HIF-1α shRNA-1 and HIF-1α shRNA-2) in knocking-down HIF-1α. When HIF-1α was knocked-down by HIF-1α shRNA-2, the enhancement of expression of *SLC2A1* (*GLUT1*) and *PGK1* by overexpression of NleB under hypoxia was diminished (Fig 5C and 5D).

To further confirm that the enhancement of hypoxia-inducible gene expression by NleB in HCT116 cells was indeed mediated by HIF-1α, we specifically inhibited HIF-1α with 25μM PX-478 [48,49]. PX-478 prevented enhanced *SLC2A1* and *PGK1* expression by overexpression of NleB under hypoxia (Fig 5E and 5F).

**Fig 6:**
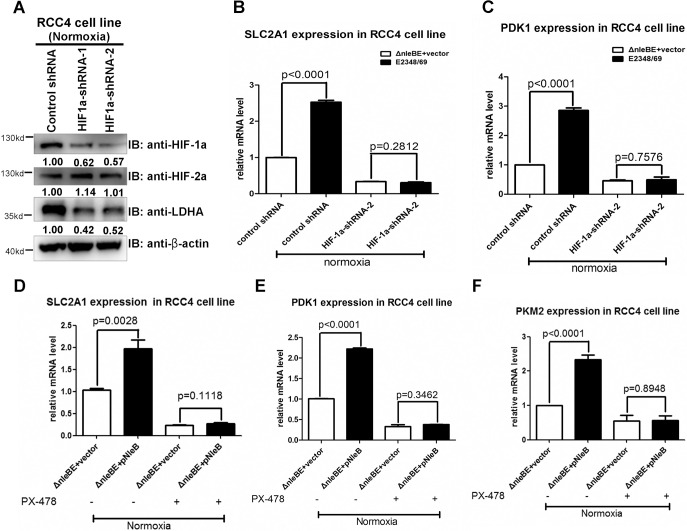
NleB-enhanced hypoxia-inducible gene expression in RCC4 cells is dependent on HIF-1α. (A) Effects of HIF-1α shRNAs (HIF-1α-shRNA-1 and HIF-1α-shRNA-2) on knockdown of endogenous HIF-1α protein in RCC4 cells. LDHA, a specific HIF-1α downstream target, was reduced when HIF-1α was knocked-down. (B, C) When HIF-1α was knocked-down by HIF-1α-shRNA-2 in RCC4 cells, the enhancement of *SLC2A1* (*GLUT1*) (B) or *PDK1* (C) expression under normoxia by infection with the wild-type EPEC strain (EPEC E2348/69) was diminished. (D, E, F) When the HIF-1α inhibitor PX-478 was added into RCC4 cells, the enhancement of *SLC2A1* (*GLUT1*) (D), PDK1 (E), or *PKM2* (F) expression by infection with the mutant EPEC strain Δ*nleBE*+pNleB was diminished (*p =* 0.1118, *p =* 0.3462, and *p =* 0.8948, respectively). Data are presented as means + SEM of three independent experiments performed in triplicate.

Furthermore, we examined the effect of NleB delivered by EPEC on hypoxia-inducible gene expression in RCC4 cells using lentivirus to knock-down HIF-1α (because RCC4 cells are difficult to transfect using the transfection reagents tested so far). As shown in Fig 6A, infection with HIF-1α shRNA lentivirus (HIF-1α-shRNA-1 and HIF-1α-shRNA-2) specifically prevented HIF-1α expression, but not HIF-2α expression, in RCC4 cells, which was further confirmed by detecting LDHA protein (a specific downstream target of HIF-1α [32]) (Fig 6A). When HIF-1α was knocked-down by adding HIF-1α-shRNA-2 lentivirus, the enhancement of expression of *SLC2A1* (*GLUT1*) and *PDK1* by infection with the wild-type EPEC E2348/69 strain was diminished (Fig 6B and 6C). Moreover, PX-478 prevented the enhanced expressions of *SLC2A1* (*GLUT1*), *PDK1*, and *PKM2* induced by infection with the mutant EPEC strain Δ*nleBE*+pNleB (Fig 6D, 6E and 6F).

Taken together, these results suggest that HIF-1α mediates NleB’s ability to enhance glucose metabolism-associated gene expression. We also confirmed these findings in HeLa cells (S7 Fig), suggesting that NleB-mediated enhancement of HIF-1α transcriptional activity was not dependent on host cell type.

Interestingly, NleB overexpression did not significantly enhance transactivity of the HIF-1α-R18K mutant (S8 Fig), reinforcing the idea that Arg18 is the key GlcNAcylated site in HIF-1α.

Given that proline hydroxylation of HIF-α by PHD proteins (PHD1, PHD2, or PHD3) heavily regulates HIF-α function [50,51], we asked whether GlcNAcylation of HIF-1α by NleB could impact HIF-1α hydroxylation. In HEK293T cells, GlcNAcylation by NleB still occurred in the hydroxylation site-dead HIF-1α mutant (DM), in which the two functional proline residues (Pro402/564) were mutated into alanine (Pro402/564Ala) (S9A Fig). Moreover, HIF-1α hydroxylation by PHD2 was unaffected by NleB expression as revealed by probing the co-IP lysates with an anti-HIF-OH antibody (S9B Fig). In addition, during EPEC infection, NleB delivered by EPEC also did not alter HIF-1α hydroxylation (S9C Fig). As expected, NleB expression in HCT116 cells also enhanced HIF-1α-DM activity under normoxia (S9D and S9E Fig).

It has been proposed that in cancer cells, OGT alters HIF-1α stability via regulation of α-ketoglutarate levels [52]. To determine whether NleB operates by a similar mechanism on HIF-1α, we measured α-ketoglutarate levels after EPEC infection. We found that NleB did not alter α-ketoglutarate levels in HCT116 cells (S10 Fig).

These results suggest that NleB enhances HIF-1α transcriptional activity, resulting in upregulation of glucose metabolism-associated genes downstream of HIF-1α.

### NleB also GlcNAcylates HIF-2α, but does not impact HIF-2α transcriptional activity

Since HIF-2α is structurally similar to HIF-1α and also performs its function under hypoxic conditions, we assessed whether NleB had a functional effect on HIF-2α in the context of hypoxia. We found that expression of wild-type NleB, but not NleB-DXD, was able to GlcNAcylate HIF-2α (S11A Fig), and NleB could also interact with HIF-2α (S11B Fig). To our surprise however, NleB had no detectable effect on the transcriptional activity of HIF-2α as revealed by promoter assays in both HCT116 and HeLa cells (S11C to S11F Fig). Actually, we had already noticed that infection with the wild-type EPEC E2348/69 strain did not enhance expression of *SOD2* (an HIF-2α-specific downstream gene) in HCT116 cells under hypoxia (Fig 4M).

Further confirming this finding, the expression of HIF-2α-specific downstream genes (*PAI1*, *POU5F1*, *SOD2*, and *CITED2*) did not change in RCC4 or 780-O (a pVHL-deficient kidney cancer cell line containing only HIF-2α) cells after infection with the EPEC strains with or without NleB [32,33], and bacteria-delivered NleB also did not change the expression of HIF-2α-specific downstream genes (S11G and S11H Fig).

Therefore, NleB may not affect HIF-2α transactivity even though it can still GlcNAcylate HIF-2α, indicating a divergent influence of NleB-mediated arginine GlcNAcylation on hypoxia signaling pathways.

### NleB-enhanced cellular glycolysis is dependent on HIF-1α, but not HIF-2α

**Fig 7:**
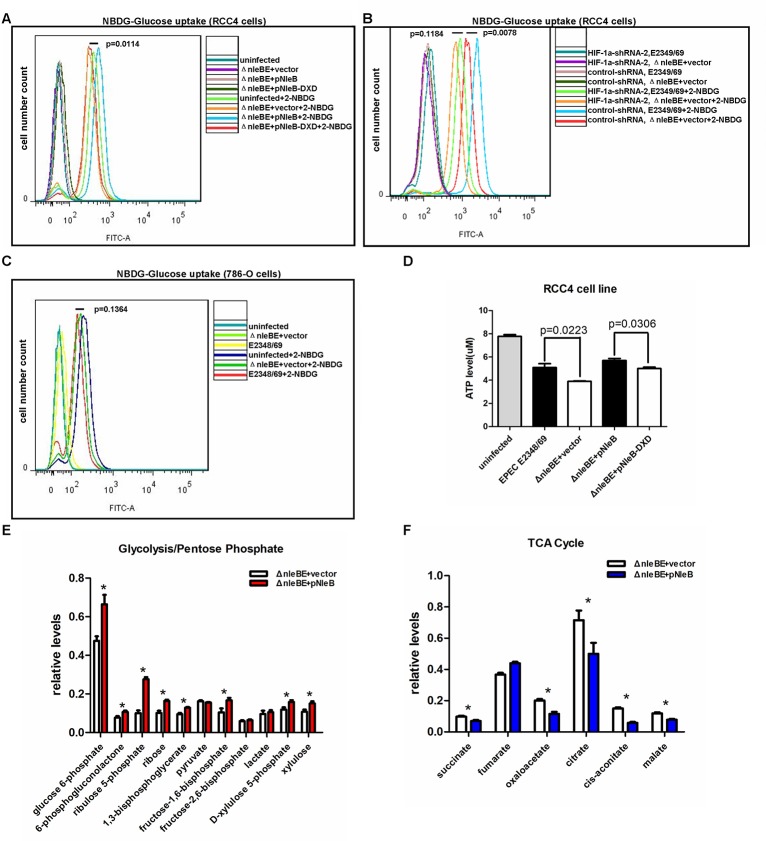
NleB enhances cellular glucose uptake, ATP levels, and glycolytic metabolism. (A) NleB caused intracellular glucose levels to significantly increase (*p =* 0.0144) in RCC4 cells. RCC4 cells were grown in the presence or the absence of the fluorescent glucose analog 2-NBDG (100 μM) for 1.5 h, after which the cells were infected with Δ*nleBE*+vector, Δ*nleBE*+NleB, Δ*nleBE*+NleB-DXD, or without infection. (B) When HIF-1α was knocked-down by HIF-1α-shRNA-2 in RCC4 cells, the enhancement of intracellular glucose level uptake by infection with the wild-type EPEC strain (EPEC E2348/69) was diminished (*p =* 0.1184). (C) In 786-O cells, infection with EPEC E2348/69 did not change intracellular glucose levels in 786-O cells (*p =* 0.1364). 786-O cells were grown in the presence or absence of 2-NBDG (100 μM) for 1.5 h, after which the cells were infected with the control EPEC strain (Δ*nleBE*+vector), the wild-type EPEC E2348/69, or without infection. Glucose uptake was quantified by flow cytometry. (D) In RCC4 cells, infection with the wild-type EPEC E2348/69 or the mutant strain Δ*nleBE*+pNleB increased ATP levels compared with cells infected with the control Δ*nleBE*+vector or the mutant EPEC strain Δ*nleBE*+pNleB-DXD, respectively (*p =* 0.0223 and *p =* 0.0306). Data are presented as means + SEM of three independent experiments performed in triplicate. (E) HCT116 cells infected with Δ*nleBE*+vector or Δ*nleBE*+pNleB respectively were collected, and levels of glycolytic/pentose phosphate pathway intermediates were measured using LC-MS. P-values were calculated using ANOVA (*p<0.05). (F) HCT116 cells infected with the control Δ*nleBE*+vector or mutant Δ*nleBE*+pNleB respectively were collected, and TCA cycle intermediates were measured using LC-MS. P-values were calculated using ANOVA (*p<0.05).

The role of HIF-1α in cellular glucose metabolism has been well defined [20,25,39,53]. Under hypoxia, HIF-1α directly transactivates the expression of glucose metabolism-associated genes [20]. Based on the above observations, NleB might enhance HIF-1α activity to upregulate the expression of glucose metabolism-associated genes, including *LDHA*, *PDK1*, *PGK1*, *SLC2A1* (*GLUT1*), *PKM2*, and *HK1*. Cellular glucose uptake is a major step in glucose metabolism and is mediated by a cell membrane glucose transport system that includes glucose transporter 1 (encoded by *GLUT1*). To further delineate the biological consequence of NleB-mediated enhancement of host glucose metabolism-associated gene expression, we examined cellular glucose uptake. In RCC4 cells (which contain both HIF-1α and HIF-2α) [30], Δ*nleBE*+pNleB infection caused higher cellular uptake of 2-NBDG (a fluorescence glucose analog) as quantified by flow cytometry (FACS) compared with cells infected with the control (*nleBE*+vector or Δ*nleBE*+pNleB-DXD strains) (*p =* 0.0114) (Fig 7A).

To further determine whether NleB-enhanced cellular glucose uptake is dependent on HIF-1α, we knocked-down HIF-1α in RCC4 cells by infection with HIF-1α shRNA-2 lentivirus. As shown in Fig 7B, when HIF-1α was knocked-down, NleB-enhanced cellular glucose uptake by infection with the wild-type EPEC E2348/69 strain was diminished (*p =* 0.1184) (Fig 7B). Additionally, in 780-O cells, which only contain HIF-2α but not HIF-1α [54], infection with the wild-type EPEC E2348/69 strain, or the control EPEC strain Δ*nleBE*+vector exhibited similar glucose uptake abilities (*p =* 0.1364) (Fig 5C).

Moreover, we examined cellular ATP levels in RCC4 cells after infection with the wild-type EPEC E2348/69 strain, the control EPEC strain Δ*nleBE*+vector, the mutant EPEC strain Δ*nleBE*+pNleB, the mutant EPEC strain Δ*nleBE*+pNleB-DXD, or without infection. As shown in Fig 7D, infection with the wild-type EPEC (EPEC E2348/69) strain enhanced cellular ATP levels compared to infection with the control EPEC strain (Δ*nleBE*+vector); infection with the mutant EPEC Δ*nleBE*+pNleB strain enhanced cellular ATP levels compared to infection with the mutant EPEC strain Δ*nleBE*+pNleB-DXD (Fig 7D), reinforcing the role of NleB in cellular glucose metabolism.

Subsequently, we used a metabolomics approach to further address whether NleB indeed enhances cellular glycolytic capacity under hypoxia. We examined the effect of NleB on intermediates from HCT116 cells using liquid chromatography-mass spectrometry (LC-MS). The metabolic profile of HCT116 cells infected by the strain ΔnleBE+pNleB demonstrated a general increase in glycolytic and pentose phosphate pathway (PPP) intermediates (Fig 7E) and a decrease in TCA cycle intermediates compared to infection with the strain ΔnleBE+vector (Fig 7F).

These results suggest that the T3SS-delivered effector NleB redirects cellular metabolism in favor of glycolysis, and that HIF-1α, but not HIF-2α, mediates the function of NleB in glucose metabolism.

### NleB enhances host glucose metabolism *in vivo*

**Fig 8:**
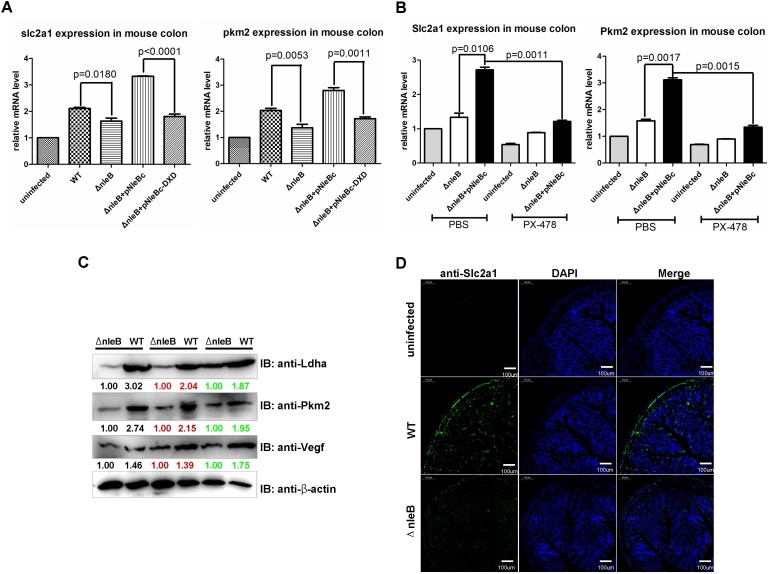
NleB enhances glucose metabolism-associated genes known to be downstream of HIF-1α *in vivo*. (A) *Slc2a1* (*Glut1*) and *Pkm2* were enhanced by NleB in mouse colon. Male C57BL/6 mice (5–6 weeks old; 17–19 g/mouse) were orally gavaged with the wild-type *C*. *rodentium* DBS100 strain, Δ*nleB* strain, or were uninfected; the mutant strain was complemented with a plasmid expressing wild-type NleB (pNleBc) or the GlcNAc transferase-deficient D221A/D223A mutant (pNleBc-DXD). After 8 days, total RNA was extracted from colons (n = 5 mice for each group), and the expressions of *Slc2a1* (*Glut1*) and *Pkm2* were examined by semi-quantitative RT-PCR. (B) Knocking-down HIF-1α by PX-478 rescued the enhancement of *Slc2a1* (*Glut1*) and *Pkm2* by NleB in mouse colon. Male C57BL/6 mice (5–6 weeks old; 17–19 g/mouse) were injected intraperitoneally with PX-478 (30 μg/g) or PBS control for 48 h, followed by oral gavage with the indicated *C*. *rodentium* strains or were uninfected. After 8 days, total RNA was extracted from colons (n = 5 mice for each group), and the expressions of *Slc2a1* (*Glut1*) and *Pkm2* were examined by semi-quantitative RT-PCR. P-values were calculated using 2-way ANOVA. (C) Protein levels of Ldha, Pkm2, and Vegf were enhanced by NleB in mouse colon. Male C57BL/6 mice (5–6 weeks old; 17–19 g/mouse) were orally gavaged with the wild-type *C*. *rodentium* DBS100 strain or Δ*nleB* strain. After 8 days, the protein levels of Ldha, Pkm2, and Vegf were examined by western blot (n = 3 mice for each group). (D) Slc2a1 (Glut1) expression was enhanced by NleB in mouse colon. Male C57BL/6 mice (5–6 weeks old; 17–19 g/mouse) were orally gavaged with the wild-type *C*. *rodentium* DBS100 strain, Δ*nleB* strain, or were uninfected. After 8 days, the expression of Slc2a1 (Glut1) was detected by immunofluorescent staining.

The above results indicate the effect of bacterial NleB on host glucose metabolism *in vitro*. To determine whether the effect on host glucose metabolism is relevant and can occur *in vivo*, we first examined the expressions of glucose metabolism-associated genes known to be downstream of HIF-1α in mouse colon after infection with wild-type *C*. *rodentium* (WT) or its mutant strains, including an *nleB-*deleted mutant (Δ*nleb*), complemented with wild-type nleB (Δ*nleb*+pNleBc), GlcNAc transferase-deficient *nleB* (Δ*nleb*+pNleBc-DXD), or without infection. The mRNA levels of *Slc2a1* (*Glut1*) and *Pkm2* were enhanced significantly after infection with wild-type *C*. *rodentium* or the mutant strain Δ*nleb*+pNleBc compared to infections with the mutant strain Δ*nleb* and the mutant strain Δ*nleb*+pNleBc-DXD (Fig 8A). However, when the mice were treated with PX-478, the enhancement of *Slc2a1* (*Glut1*) and *Pkm2* by NleB in mouse colon was diminished (Fig 8B). Moreover, the protein levels of Ldha, Pkm2, and Vegf (another downstream target of HIF-α irrelevant to glucose metabolism) were also higher in mouse colons infected with *C*. *rodentium* WT compared to those infected with the *nleB-*deleted mutant, Δ*nleb* (Fig 8C). By immunofluorescent staining, Slc2a1 (Glut1) was found to be more highly expressed in mouse colons infected with *C*. *rodentium* WT compared to those infected with Δ*nleb* (Fig 8D).

To validate the effect of PX-478 on the suppression of HIF-1α expression in mouse colon, we examined the protein levels of HIF-1α by western blotting (S12A Fig). Treatment with PX-478 also inhibited the expressions of glucose metabolism-associated genes known to be downstream of HIF-1α in mouse colons (S12B Fig). Notably, PX-478 did not alter the colonization of *C*. *rodentium* in feces and mouse colon (S12C and S12D Fig).

Furthermore, we examined blood glucose levels in mice after infection with the *C*. *rodentium* WT or its mutant strains, including Δ*nleb*, complemented with wildtype nleB (Δ*nleb*+pNleBc) or GlcNAc transferase-deficient *nleB* (Δ*nleb*+pNleBc-DXD). At timepoints either before or after fasting (6 h), mice infected with *C*. *rodentium* WT had significantly lower blood glucose levels compared to mice infected with the Δ*nleb* mutant strain (S13A and S13B Fig), and we confirmed that GlcNAc transferase activity was required for nleB to reduce mouse blood glucose levels (S13A and S13B Fig). Notably, the blood glucose levels in mice infected with the Δ*nleb*+pNleBc strain were lower than those in mice infected with *C*. *rodentium* WT (S13A and S13B Fig), implying that the Δ*nleb* pNleBc strain could deliver more pNleBc into host cells than *C*. *rodentium* WT due to overexpression of pNleBc, resulting in greater enhancement of HIF-1α activity. In addition, this effect also depended on Hif-1α activity, as the inhibitory role of nleB on mouse blood glucose levels diminished when the mice were treated with PX-478 (S13A and S13B Fig).

Given that the liver is virtually the only major organ that produces and supplies blood glucose to maintain blood glucose levels, we tested if gastrointestinal infection with *C*. *rodentium* leads to glucose metabolism-associated gene expression alterations in the liver in addition to their actual infection site—the colon. We went on to examine *slc2a1* and *pkm2* expression levels in mouse livers after infection with the different *C*. *rodentium* strains. Consistent with the role of NleB in the colon and on blood glucose levels, NleB enhanced glucose metabolism-associated gene expression, and GlcNAc transferase activity was required for this enhancement (S13E and S13F Fig). The reduction of Hif-1α by PX-478 injection was confirmed in mouse livers by western blot (S13G Fig). However, NleB had no effect on the proliferation of pancreatic islets and did not alter blood insulin levels in mice (S14A and S14B Fig). Moreover, NleB also did not change homeostasis model assessment of insulin resistance (HOMA-IR) significantly in mice (S14C Fig). It appears that NleB affects glucose metabolism independent of host insulin action.

These results suggest that NleB enhances host glucose metabolism *in vivo* and that NleB requires its GlcNAc transferase activity to function efficiently.

## Discussion

Accumulating evidence has revealed that pathogens affect energy homeostasis, glucose metabolism, and metabolic inflammation, but the causal link between pathogen infection and host metabolism remains to be fully understood [10,55,56]. In this study, our finding that bacterial NleB, a virulence protein (effector) delivered by the T3SS in gut A/E pathogens such as EPEC or *C*. *rodentium*, modulates host glucose metabolism by GlcNAcylating arginine residues in HIF-1α [13] provides a causal link between pathogen infection and host glucose metabolism; furthermore, this finding could open new avenues for exploring the underlying causes by which pathogen infection affects host metabolism. Given that the regulation of host glucose metabolism is complicated and that HIF-1α mainly participates in glucose metabolism under hypoxia [20], future studies determining how pathogens modulate host glucose metabolism beyond HIF-1 signaling would be of great interest.

It has been noticed that NleB overexpression either by direct transfection of expression vectors or by infection with the mutant strain Δ*nleBE*+pNleB might GlcNAcylate targets non-specifically [57]. Therefore, we must seriously consider whether HIF-1α is a specific target modified by NleB. In this study, even though we could not always detect the GlcNAcylation of endogenous HIF-1α by infection of the wild-type EPEC strain, we always observed that NleB delivered by the wild-type strain enhanced HIF-1α transcriptional activity. In addition, knockdown of HIF-1α by HIF-1α shRNA diminished the enhancement of glucose uptake by NleB delivered by the wild-type EPEC strain. Furthermore, knockdown of HIF-1α either by HIF-1α shRNA or by the inhibitor PX-478 diminished the enhancement effect of NleB on HIF-1α transcriptional activity. Thus, it appears that HIF-1α is a native target of NleB. Because of the relatively low level of NleB delivered by the wild-type EPEC strain compared to that delivered by the mutant strain Δ*nleBE*+pNleB in which NleB is overexpressed, it was often hard to detect GlcNAcylation of endogenous HIF-1α by western blotting. In addition, in the mouse infection model, we noticed that infection with Δ*nleB*+pNleBc resulted in lower levels of blood glucose compared to infection with *C*. *rodentium* WT. The culture status of the wild-type EPEC strain and/or host cells might affect the efficiency of NleB GlcNAcylation of HIF-1α.

Remarkably, the HIF-1α-R18K mutant was still GlcNAcylated by NleB even though it exhibited the lowest GlcNAcylation efficiency compared to all other mutants, indicating that arginine residues other than arginine 18 in HIF-1α might still be GlcNAcylated by NleB. However, NleB did not significantly enhance the transactivity of the HIF-1α-R18K mutant. Therefore, R18 at the N-terminus of HIF-1α appears to be the key site for GlcNAcylation by NleB. Of note, R18 is located in the DNA binding domain (DB), but not the transactivation domain (TAD), of HIF-1α. So GlcNAcylation of HIF-1α protein by NleB might mainly enhance the DNA binding ability of HIF-1α rather than enhancing transactivity directly, resulting in enhanced hypoxia-inducible glucose metabolism-associated gene expression.

However, due to technical limitations, we could not provide direct evidence that endogenous HIF-1α was indeed GlcNAcylated by NleB after infection with the WT and nleB mutant. Therefore, we are not sure whether HIF-1α is modified by NleB under physiological levels of the effector. Hopefully, more advanced techniques will be developed to specifically address this concern and clarify it in the near future.

Pathogens also affect host glucose metabolism via various mediators encoded in pathogens and host cells [58–63], raising the question of whether the host, the pathogen, or both benefit from the interaction. On one hand, modulation of host glucose metabolism by the pathogen might benefit pathogen infection [63–65]. On the other hand, this modulation might also contribute to the development of host diseases related to glucose metabolic disturbance. Therefore, further investigation into the relationship between pathogen infection and host glucose metabolism may provide some clues for developing strategies for both treatment of pathogen infection and the prevention of host diseases related to disturbances in glucose metabolism. Interestingly, normal glucose uptake in the brain and heart requires an endothelial cell-specific HIF-1α-dependent function, which is consistent with our observation that HIF-1α enhancement by NleB increases glucose uptake [25].

As a virulence protein, NleB is known to influence the host immune response, such as through its effects on FADD, TRADD, and RIPK1 [11,13,14,16]. Since HIF-1α is also involved in host immune response to bacterial infection [66–69], we initially also asked whether NleB affected the host immune response by modifying HIF-1α. However, we could not activate an NF-kb reporter and induce TNF-α expression by overexpressing HIF-1α in our cellular system, which should have worked based on previous reports [66,67]. Therefore, we still cannot definitively answer whether NleB affects host immune response through modification of HIF-1α.

Intriguingly, even though NleB GlcNAcylated arginine(s) on HIF-2α, NleB did not enhance HIF-2α transcriptional activity like it did for HIF-1α. As two master regulators of hypoxia signaling, HIF-1α and HIF-2α share the common function of regulating similar downstream genes, but HIF-1α and HIF-2α also have divergent functions regulating their own specific downstream targets [39]. Thus far, since HIF-1α, but not HIF-2α, has been identified as a regulator of glucose metabolism [20], the fact that NleB specifically enhanced HIF-1α transactivity is consistent with its role in host glucose metabolism. Nevertheless, the consequences of NleB GlcNAcylating arginine(s) in HIF-2α are of interest for future studies.

Due to embryonic lethality of HIF-1α-null mice [70,71], we cannot provide genetic evidence to show that NleB-enhanced host glucose metabolism is indeed mediated by HIF-1α. In this study, to obtain *in vivo* data to understand the physiological role of the type III effector NleB, we used the HIF-1α inhibitor PX-478 in a mouse model. Even though there are non-specific inhibitory roles of PX-478, we found that PX-478 had the same effect as that of HIF-1α shRNA in the cell culture system. In addition, PX-478 effectively blocked HIF-1α protein levels in mouse colon and liver. Therefore, PX-478 might be suitable for specifically suppressing HIF-1α function in mouse models.

**Fig 9:**
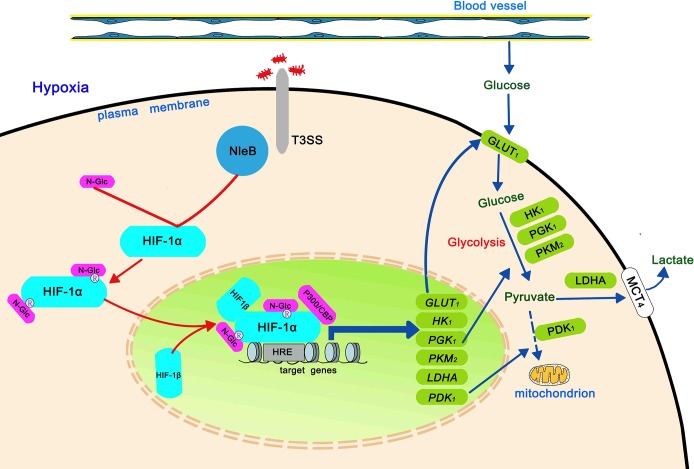
Working model for the role of NleB in host glucose metabolism. Host intestinal epithelial cells maintain low O_2_ conditions due to counter-current blood flow. When bacteria such as EPEC infect intestinal epithelial cells, the T3SS delivers NleB into them, then NleB binds and GlcNAcylates HIF-1α at arginine residues, enhancing HIF-1α transcriptional activity and increasing the expression of downstream glucose metabolism-associated gene targets to modulate processes related to glucose uptake (*GLUT1*) and glycolysis (*HK1*, *PGK1*, *PKM2*, *LDHA*, and *PDK1*).

Based on the observations in this study, we propose a working model for the role of bacteria-delivered NleB on host glucose metabolism via its effect on HIF-1α (Fig 9). The host gut maintains low O_2_ conditions (hypoxia) due to counter-current blood flow [28]. When bacteria such as EPEC infect the intestines, the T3SS delivers NleB into host intestinal cells. NleB then binds and GlcNAcylates HIF-1α at arginine residues, enhancing HIF-1α transcriptional activity and increasing expression of glucose metabolism-associated genes downstream of HIF-1α. This modulates processes related to glucose uptake (*GLUT1*) and glycolysis (*HK1*, *PGK1*, *PKM2*, *LDHA*, and *PDK1*).

## Materials and methods

### Ethics statement

All procedures involving the use of mice were approved by the ethical board of the Animal Care and Use Committee of the Institute of Hydrobiology, Chinese Academy of Sciences (protocol number IHB-2017001). All protocols conform to the Guide for the Care and Use of Laboratory Animals of the U.S. National Institutes of Health. All efforts were made to minimize the number of animals and their suffering.

### Cell culture

HEK293T, HeLa, HCT116, and 786-O cells were originally obtained from the American Type Culture Collection (ATCC). RCC4 cells were kindly provided by Peter J. Ratcliffe. HEK293T, HeLa, and RCC4 cells were grown in Dulbecco’s modified Eagle’s medium (DMEM) (HyClone) supplemented with 10% fetal bovine serum (FBS). 786-O cells were grown in RPMI 1640 (HyClone) supplemented with 10% FBS. HCT116 cells were grown in Mc-Coy5A (HyClone) supplemented with 10% FBS. Cells were cultivated in a humidified incubator containing 5% CO_2_ at 37°C. Cells were cultivated under hypoxic conditions (2% O_2_) by using an incubator with O_2_ control filled with 5% CO_2_ and balanced with N_2_ (NBS Galaxy 48R). All cell lines were verified to be free of *Mycoplama* contamination before use.

### Luciferase reporter assays

Cells were seeded in 24-well plates and transfected with the indicated plasmids using VigoFect (Vigorous Biotech, Beijing, China). pRL-SV40 *Renilla* was as an internal control. Luciferase activity was measured 18–24 h after transfection using the Dual-luciferase Reporter Assay System (Promega). Data were normalized to *Renilla* luciferase. Luciferase data are reported as mean ± s.e.m. of three independent experiments performed in triplicate.

### Bacterial strains and cell culture infection

The EPEC E2348/69 ∆nleB/E SC3909 strain (∆IE2:: kan and nleBE IE6:: tet) and its derivatives were kindly provided by Feng Shao, and were used for cell culture infection (S1 Table). A single bacterial colony was inoculated into 0.5 mL of LB medium and statically cultured overnight at 37°C. Bacterial cultures were then diluted 1:40 in DMEM supplemented with 1 mM isopropyl-β-D-thiogalactoside (IPTG) and cultured for an additional 4 h at 37°C in the presence of 5% CO_2_. Infection was performed at a multiplicity of infection of 200:1 in the presence of 1 mM IPTG for 2 h. Cells were washed four times with PBS. To assay NleB-induced modification, HEK 293T cells were transfected with pCMV-Myc-HIF-1α or pCMV-Myc-HIF-1α plasmids 24 h before infection.

### Plasmid construction

The p2.1 reporter was purchased from ATCC and is commonly used for monitoring HIF-1α transcriptional activity [34]. Hypoxia response element (HRE) reporter and SOD2 promoter luciferase reporter were provided by Navdeep Chandel and Xin-Hua Feng, respectively. The HRE-reporter contains three hypoxia response element (HRE) repeats and is commonly used for monitoring HIF-1α transcriptional activity [35]. The wild-type EPEC NleB gene was originally obtained from Feng Shao, and its enzymatically dead mutant (D221/223A) was PCR amplified and subcloned into the pCS2-EGFP vector and pGEX-4T vector. Wild-type human HIF-1α was subcloned into the pCMV-Myc vector and pCMV-HA vector (Clontech). Human HIF-1α-330-399aa was cloned into the pET32a vector. All R/K mutants of HIF-1α were subcloned into the pCMV-Myc vector. Wild-type human HIF-2α was subcloned into the pCMV-Myc vector and the pCMV-HA vector. Human HIF-1α domains were subcloned into the pCMV-Myc vector.

### Antibodies and reagents

The antibodies used were as follows: anti-c-Myc antibody (9E10, 1:1000 for IB analysis, Santa Cruz), anti-HA antibody (1:5000 for IB analysis, Covance), anti-β-actin antibody (AC004, 1:20,000 for IB analysis, ABclonal), anti-HIF-1α antibody (A6265, 1:1000 for IB analysis, ABclonal), anti-GFP antibody (AE012, 1:1000 for IB analysis, ABclonal), anti-HIF-OH antibody (3434S, 1:1000 for IB analysis, Cell Signaling), anti-Arginine (glcnac) (ab195013, 1:1000 for IB analysis, Abcam), anti-His (H15, 1:500 for IB analysis, Santa Cruz), anti-Ldha antibody (A1146, 1:1000 for IB analysis, ABclonal), anti-Pkm2 antibody (GB11392, 1:1000 for IB analysis, Servicebio), anti-Vegf (GB11034, 1:1000 for IB analysis, Servicebio), anti-Slc2a1 (Glut1) antibody (GB11215, 1:100 for IF analysis, Servicebio), anti-Ki67 antibody (GB113030-2, 1:100 for IF analysis, Servicebio), anti-Insulin antibody (GB13121, 1:100 for IF analysis, Servicebio).

PX-478 (S7612) was purchased from Selleck. 6-[N-(7-Nitrobenz-2-oxa-1,3-diazol-4-yl) amino]-2-deoxy-D-glucose (2-NBDG), a glucose analog, was purchased from Invitrogen. Insulin (Mouse) ELISA Kit (KA3812) was purchased from Abnova. Alpha-Ketoglutarate Assay Kit (K677-100) was purchased from Biovision.

### Immunoprecipitation and western blot

Coimmunoprecipitation and western blot analysis were performed as previously described [33]. Anti-Myc and anti-Flag antibody-conjugated agarose beads were purchased from Sigma. Protein A/G-Sepharose beads were purchased from GE Company. A Fuji Film LAS4000 mini-luminescent image analyzer was used to photograph the blots. Multi Gauge V3.0 was used to quantify protein levels based on the band density.

### GST pulldown assays

His-tagged HIF-1α-330aa-399aa and GST-tagged NleB (DXD) were expressed in *E*. *coli* BL21-Gold (DE3). GST resin (Novagen) was used for protein purification. The gels were stained by Coomassie blue or transferred to polyvinylidene difluoride (PVDF) membranes for western blot assays.

### Semi-quantitative real-time PCR

Total RNA was extracted using TRIzol reagent (Invitrogen). cDNA was synthesized using a first-strand cDNA synthesis kit (Fermentas). The following primers were used for internal control 18S rRNA: 5’-TCAACTTCGATGGTAGTCGCCGT-3’ and 5’-TCCTTGGATGTGGTAGCCGTTCT-3’. Other primers were synthesized as described in previous reports [32,33]. Semi-quantitative RT-PCR data are reported as mean ± s.e.m. of three independent experiments performed in triplicate.

### Mass spectrum analysis

To determine GlcNAcylation site(s) in HIF-1α by NleB, HA-HIF-1α and GFP-NleB were co-expressed in 293T cells, and HA-HIF-1α was purified using anti-HA antibody-conjugated agarose beads. Then, the purified HIF-1α was digested by trypsin and analyzed by online nanoflow LC-MS/MS using the Ultimate 3000 nano-LC system (Dionex, Sunnyvale, CA) connected to an LTQ-Orbitrap Elite (Thermo Scientific) mass spectrometer. Samples were injected onto an analytical C18-nanocapillary LC column (C18 resin with 3 μm particle size, 15 cm length × 75 μm inner diameter, Acclaim PepMap RSLC, Thermo Scientific) and eluted at a flow rate of 300 nL/min with a 130 min gradient from 5% solvent B (90% ACN/0.1% formic acid, v/v) to 50% solvent B. The peptides were then directly ionized and sprayed into an Orbitrap Elite mass spectrometer by a nanospray ion source. The mass spectrometer was operated in data-dependent mode with an automatic switch between MS and MS/MS acquisition. Full MS spectra from *m/z* 350 to 1800 were acquired with a resolution of 60,000 at *m/z* 400 in profile mode. Following every survey scan, up to 15 of the most intense precursor ions were picked for MS/MS fragmentation by high-energy collisional dissociation (HCD) with a normalized collision energy of 30%. The dynamic exclusion duration was set to 120 s with a repeat count of one and ±10 ppm exclusion window.

All acquired raw data were processed with pFind software (version 3.1.2) (Chi et al., 2018) and searched against the NCBI Human Protein Database (https://www.ncbi.nlm.nih.gov/protein). Two missed cleavages were allowed for trypsin. The precursor and fragment ion mass tolerances were set to 20 ppm. Carbamidomethyl (C) was set as a fixed modification, whereas N-acetylhexosamine addition to arginine (Arg-GlcNAc), deamidation (NQ), and oxidation (M) were selected as variable modifications. The estimated false discovery rate (FDR) of peptide identification was less than 1%.

### Metabolite analysis

HCT116 cells were cultured under hypoxia for 12 h, and then infected with the bacterial strain ΔnleBE+vector or ΔnleBE+pNleB for 4 h. Cells were counted, and approximately 10^**7**^ cells per sample were spun down at 300 *g* for 3 min. Cells were washed twice with PBS and then snap-frozen on dry ice and stored at −80°C until analysis by Metabolon. After the addition of 1000 μL of extract solvent (acetonitrile-methanol-water, 2:2:1, containing internal standard), the samples were vortexed for 30 s, homogenized at 45 Hz for 4 min, and sonicated for 5 min in an ice-water bath. The homogenization and sonication cycle were repeated 3 times, followed by incubation at −20°C for 1 h and centrifugation at 12,000 rpm (4°C) for 15 min. The resulting supernatants were transferred to LC-MS vials and stored at −80°C until UHPLC-QE Orbitrap/MS analysis. The quality control (QC) sample was prepared by mixing an equal aliquot of the supernatants from all of the samples. LC-MS/MS analyses were performed using an UHPLC system (1290, Agilent Technologies) with a UPLC HSS T3 column (2.1 mm × 100 mm, 1.8 μm) coupled to Q Exactive (Orbitrap MS, Thermo). The mobile phase A was 0.1% formic acid in water for positive, and 5 mmol/L ammonium acetate in water for negative, and the mobile phase B was acetonitrile. The elution gradient was set as follows: 0 min, 1% B; 1 min, 1% B; 8 min, 99% B; 10 min, 99% B; 10.1 min, 1% B; 12 min, 1% B. The flow rate was 0.5 mL/min. The injection volume was 3 μL. The QE mass spectrometer was used for acquiring MS/MS spectra on an information-dependent basis (IDA) during LC/MS experiments. In this mode, the acquisition software (Xcalibur 4.0.27, Thermo) continuously evaluates the full scan survey MS data. ESI source conditions were set as follows: sheath gas flow rate as 45 Arb, Aux gas flow rate as 15Arb, capillary temperature 320°C, full ms resolution as 70,000, MS/MS resolution as 17,500, collision energy as 20/40/60 eV in NCE mode, spray voltage as 3.8 kV (positive) or −3.1 kV (negative), respectively.

### Glucose uptake assays

RCC4 and 786-O cells were infected with EPEC E2348/69 and it derivatives for 2–4 h. RCC4 or 786-O cells were grown under normal conditions with or without 100 μM 2-NBDG (Invitrogen) for 1.5 h. Fluorescence was measured with a fluorescence-activated cell sorting (FACS) analyzer.

### Gene knockdown

Control short hairpin RNA (luciferase shRNA) and HIF-1α short hairpin RNAs (HIF-1α shRNA-1 and HIF-1α shRNA-2) were cloned into the lentivirus vector lentiLox3.7. The shRNA target sequences were as follows: control shRNA (luciferase shRNA), 5’-GTTGGCACCAGCAGCGCAC-3’; HIF-1α shRNA-1, 5’-GAGCTTGCTCATCAGTTGC-3’; HIF-1α shRNA-2, 5’-GGGTTGAAACTCAAGCAAC-3’.

For knocking-down HIF-1α in HCT116 cells, control shRNA vector, HIF-1α shRNA-1 vector, or HIF-1α shRNA-2 vector were transiently transfected into HCT116 cells respectively. For knocking-down HIF-1α in RCC4 cells, due to the extreme low efficiency for transfection in RCC4 cells, lentiviruses were produced. Lentivirus of HIF-1α shRNAs and control (luciferase) shRNA were generated by transfecting HEK293T cells with transducing vector packaging vectors VSVG, RSV-REV, and pMDL g/p RRE together with control shRNA vector, HIF-1α shRNA-1 vector, or HIF-1α shRNA-2 vector. After transfection for 48 h, virus particles in the medium were harvested, filtered, and transduced into target cells.

### ATP concentration assays

RCC4 cells and 786-O cells were transduced with lentivirus encoding control shRNA or HIF-1α shRNAs in the absence or presence of bacterial strains for 3 h, and ATP concentrations were measured by an ATP assay kit (Beyotime) following the manufacturer’s instructions.

### Animal Experiments

Male C57BL/6 mice, 5–6 weeks old, were individually maintained in high-efficiency particulate air (HEPA)-filtered cages with autoclaved food and water. Mice were randomized into each experimental group without investigator blinding. All mice were male and of the same size distribution (17–19 g).

Deletion of the gene encoding NleBc in *C*. *rodentium* strain DBS100 (ATCC51459; ATCC) and its derivatives were provided by Feng Shao (S1 Table). For oral inoculation, *C*. *rodentium* wild-type strain and its derivatives were prepared by shaking the bacterial culture overnight at 37°C in LB broth. Mice were orally inoculated using a gavage needle with 200 μl bacterial suspension in PBS (~4 × 10^9^ CFU or ~2 × 10^9^ CFU).

Mice were weighed every 2 days and feces collected every 2 or 4 days for enumeration of CFU (~2 × 10^9^ CFU), and the number of viable bacteria per gram of feces was determined by plating serial dilutions onto LB agar containing the appropriate antibiotics. Eight days after inoculation, colons were removed aseptically, weighed, and diluted in PBS. The serial dilutions were plated to determine CFU counts. Colonization data were analyzed using Student’s *t* test (GraphPad Prism 5.0). P < 0.05 was considered significant.

For western blot assays of mouse colon, the distal portion of the colon from the caecum to the rectum was removed from infected mice. For immunofluorescent staining of mouse colon, colon sections were incubated with anti-Slc2a1(glut1) antibody diluted in PBS (1:100).

For PX-478 treatment, PX-478 powder (Selleck) was dissolved in PBS (20 μg/μl, stock solution). The stock solution was diluted to 1.2 μg/μL (working solution), and a total of 500 μL of the working solution (600 μg/each mouse) was injected intraperitoneally (30 μg/g; final injected dosage for each mouse was based on a 20 g body weight). Two days after PX-478 injection, mice were orally inoculated using a gavage needle with 200 μL bacterial suspension in PBS (~4 × 10^9^ CFU). Pre- and post-fasting (6 h) blood glucose was measured.

Two days after inoculation of bacteria (~4 × 10^9^ CFU), blood glucose was obtained by tail bleeding before fasting, and glucose was measured using an automatic glucose monitor (Ultra One Touch) (before fasting). Then, mice were fasted for 6 h and blood glucose was measured again after fasting. Two independent experiments were performed using 3 or 4 mice per group.

### Statistical analysis

All independent experiments carried out in this study and indicated in the figure legends were biological replicates. All results are presented as means + SEM. Unless otherwise noted, p-values were calculated using unpaired *t* tests (GraphPad Prism 5, GraphPad Software Inc.).

## Supporting information

S1 Table Bacterial strains used in this study. (DOC)

S2 Table Summary of observed modifications in HIF-1α by NleB as revealed by MS/MS analysis. (DOC)

S1 Fig Endogenous HIF-1α in RCC4 cells is GlcNAcylated by T3SS-delivered NleB. RCC4 cells were uninfected or infected with the wild-type EPEC strain (EPEC E2348/69); a mutant EPEC strain lacking *escN* (indicated as Δ*escN*) but complemented with a plasmid expressing WT NleB (Δ*escN* + pNleB); or a mutant EPEC strain lacking both *nleE* and *nleB* (strain SC309, indicated as Δ*nleBE*) but complemented with an empty plasmid (Δ*nleBE* + vector), a plasmid expressing wild-type NleB (Δ*nleBE*+pNleB), ora plasmid expressing the GlcNAc transferase-deficient D221A/D223A mutant (Δ*nleBE*+pNleB-DXD). The cell lysates were subjected to anti-HIF-1α IP and detected by an anti-arginine (GlcNAc) antibody. IP, immunoprecipitation; TCL, total cell lysates; IB, immunoblotting.
(TIF)

S2 Fig NleB interacts with the HIF-1α-R-free mutant, and the GlcNAc transferase-deficient NleB interacts with wild-type HIF-1α. (A) NleB interacted with the HIF-1α-R-free mutant. (B) The GlcNAc transferase-deficient NleB, NleB-DXD, interacted with wild-type HIF-1α**.** HEK293T cells were transfected with the indicated plasmids. Anti-Myc antibody-conjugated agarose beads were used for co-IP, and anti-GFP antibody was used for detection. In HIF-1α-R-free, all 35 arginine residues in human HIF-1α were simultaneously mutated to lysine residues; NleB-DXD, NleB Asp221Ala/Asp223Ala double mutant; IP, immunoprecipitation; TCL, total cell lysates; GFP-NleB, GFP-tagged wild-type NleB.
(TIF)

S3 Fig Protein expression detected in HCT116 cells. (A) Expressions of HIF-1α and NleB were confirmed in HCT116 cells after transfection for promoter assays (Fig 4A). (B) Expressions of HIF-1α and NleB were confirmed in HCT116 cells after transfection for promoter assays (Fig 4B). (C) Expression of NleB was confirmed in HCT116 cells after transfection for promoter assays (Fig 4C). (D) Expression of NleB was confirmed in HCT116 cells after transfection for promoter assays (Fig 4D). (E)Expression of NleB was confirmed in HCT116 cells after transfection for RT-PCR assays (Fig 4E–4H). (F) The levels of NleB and NleB-DXD in HCT116 cells translocated from the EPEC mutant Δ*nleBE*+HA-pNleB and the EPEC mutant Δ*nleBE*+HA-pNleB-DXD respectively as revealed by western blot assays using anti-HA antibody.
(TIF)

S4 Fig NleE enhances HIF-1α transcriptional activity. (A, B) Induction of HRE-reporter luciferase activity (A) or p2.1-reporter luciferase activity (B) under hypoxia was significantly enhanced by NleB transfection in HCT116 cells (*p =* 0.0050 and *p =* 0.0002, respectively). (C, D) Induction of HRE-reporter luciferase activity (C) or p2.1-reporter luciferase activity (D) under hypoxia was significantly enhanced by NleB transfection in HeLa cells (*p =* 0.0032 and *p =* 0.0004, respectively).
(TIF)

S5 Fig NleB enhances BNIP3 promoter activity activated by HIF-1α. (A, B) Induction of BNIP-reporter luciferase activity by HIF-1α transfection was significantly enhanced by NleB transfection in HCT116 cells or HeLa cells (*p =* 0.0006 and *p =* 0.0013, respectively). (C, D) Induction of BNIP-reporter luciferase activity by HIF-1α transfection was significantly enhanced by infection with the wild-type EPEC strain (EPEC E2348/69) compared to infection with the mutant EPEC strain lacking both *nleE* and *nleB* (strain SC309) but complemented with an empty plasmid (Δ*nleBE*+vector) in HCT116 cells or HeLa cells (*p =* 0.0102 and *p =* 0.0383, respectively).
(TIF)

S6 Fig Effects of HIF-1α shRNAs (HIF-1α-shRNA-1 and HIF-1α-shRNA-2) on knockdown of endogenous HIF-1α protein in HCT116 cells. (TIF)

S7 Fig NleB enhances HIF-1α transcriptional activity in HeLa cells. (A, B) Induction of HRE-reporter luciferase activity (A) or p2.1-reporter luciferase activity (B) by HIF-1α transfection under normoxia was significantly enhanced by NleB transfection in HeLa cells (*p =* 0.0033 and *p =* 0.0021, respectively). HRE, hypoxia response element. (C, D) Induction of HRE-reporter luciferase activity (C) or p2.1-reporter luciferase activity (D) under hypoxia was significantly enhanced by NleB transfection in HeLa cells (*p =* 0.0002 and *p =* 0.0144, respectively). (E, F, G, H) Induction of *LDHA* (E), *HK1*(F), *PDK1* (G), or *PGK1* (H) mRNA expression under hypoxia was significantly enhanced by NleB transfection in HeLa cells (*p =* 0.0033, 0.0003, 0.0077, and 0.0035, respectively). (I, J) Induction of HRE-reporter luciferase activity (I) or p2.1-reporter luciferase activity (J) by HIF-1α transfection under normoxia in HeLa cells was significantly enhanced by infection with the wild-type EPEC strain (EPEC E2348/69) compared to infection with the mutant EPEC strain lacking both *nleE* and *nleB* (strain SC309) but complemented with an empty plasmid (Δ*nleBE*+vector) (*p =* 0.0039 and *p =* 0.0009, respectively). (K, L) Induction of HRE-reporter luciferase activity (K) or p2.1-reporter luciferase activity (L) in HeLa cells infected with EPEC E2348/69 was significantly enhanced under hypoxia (p<0.0003 and *p =* 0.0133, respectively). (M, N) The HIF-1α inhibitor PX-478 (25μM) blocked the enhancement of *SLC2A1* (M) or *PDK1* (N) mRNA expression by NleB transfection under hypoxia in HeLa cells (*p =* 0.4410 and *p =* 0.3177, respectively). Data are presented as means + SEM of three independent experiments performed in triplicate.
(TIF)

S8 Fig NleB does not significantly enhance HIF-1α (R18K) mutant transcriptional activity. (A) Induction of HRE-reporter luciferase activity by HIF-1α (R18K) mutant transfection under normoxia was not significantly enhanced by NleB transfection in HCT116 cells (*p =* 0.0815) compared with that by wild-type HIF-1α (*p =* 0.0032). HRE, hypoxia response element. (B) Induction of p2.1-reporter luciferase activity by HIF-1α (R18K) mutant transfection under normoxia was not significantly enhanced by NleB transfection in HCT116 cells (*p =* 0.1425) compared with that by wild-type HIF-1α (*p =* 0.0014). Data are presented as means + SEM of three independent experiments performed in triplicate.
(TIF)

S9 Fig Arginine GlcNAcylation of HIF-1α by NleB has no effect on hydroxylation of HIF-1α by PHD2. (A) Effects of NleB transfection on arginine GlcNAcylation of the hydroxylated site-mutated HIF-1α (DM) in HEK293T cells. IP, immunoprecipitation; TCL, total cell lysates; GFP-NleB, GFP-tagged wild-type NleB; WT, wild-type HIF-1α; DM, a HIF-1α mutant with two proline residues mutated to alanine residues (P402A/P564A). (B) Effects of NleB transfection on hydroxylation of HIF-1α by PHD2 in HEK293T cells. (C) Effects of NleB on hydroxylation of endogenous HIF-1α in HCT116 cells after infection with the indicated EPEC strains under either normoxia or hypoxia. (D) Induction of HRE-reporter luciferase activity by HIF-1α-DM transfection under normoxia was significantly enhanced by NleB transfection in HCT116 cells (*p =* 0.0011). HRE, hypoxia response element. (E) Induction of p2.1-reporter luciferase activity by HIF-1α-DM transfection under normoxia was significantly enhanced by NleB transfection in HCT116 cells (*p =* 0.0040). Data are presented as means + SEM of three independent experiments performed in triplicate.
(TIF)

S10 Fig Cellular α-ketoglutarate levels are not affected by NleB. (A) Cellular α-ketoglutarate levels in HCT116 cells were measured after infection or without infection with mutant EPEC strains lacking both *nleE* and *nleB* (strain SC309, indicated as Δ*nleBE*) but complemented with an empty plasmid (Δ*nleBE*+vector) or a plasmid expressing wild-type HA-tagged NleB (Δ*nleBE*+HA-pNleB) for 4 h under normoxia or hypoxia (the cells were pre-treated under hypoxia for 12 h before infection). (B) Expression of HA-NleB was confirmed by western blot.
(TIF)

S11 Fig NleB GlcNAcylates the HIF-2α protein but does not affect HIF-2α transcriptional activity. (A) Effects of NleB transfection on arginine GlcNAcylation of HIF-2α in HEK293T cells. IP, immunoprecipitation; TCL, total cell lysates; GFP-NleB, GFP-tagged wild-type NleB; GFP-BleB-DXD, GFP-tagged NleB Asp221Ala/Asp223Ala double mutant. (B) NleB interacted with HIF-2α. HEK293T cells were transfected with the indicated plasmids. Anti-Myc antibody-conjugated agarose beads were used for co-immunoprecipitation, and anti-GFP and anti-Myc antibodies were used for detection. (C) Induction of HRE-reporter luciferase activity by HIF-2α transfection under normoxia was not affected by NleB transfection in HCT116 cells (*p =* 0.6312). HRE, hypoxia response element. (D) Induction of *SOD2*-reporter luciferase activity by HIF-2α transfection under normoxia was not affected by NleB transfection in HCT116 cells (*p =* 0.4710). (E) Induction of HRE-reporter luciferase activity by HIF-2α transfection under normoxia was not affected by NleB transfection in HeLa cells (*p =* 0.7753). (F) Induction of *SOD2*-reporter luciferase activity by HIF-2α transfection under normoxia was not affected by NleB transfection in HeLa cells (*p =* 0.2747). (G) Expression of HIF-2α target genes *PAI1*, *POU5F1*, and *SOD2* in RCC4 cells was not affected by bacteria-delivered NleB. RCC4 cells were infected with the mutant EPEC strains Δ*nleBE*+vector or Δ*nleB*+pNleB, respectively; semi-quantitative RT-PCR was used to detect mRNA levels of *PAI1*, *POU5F1*, and *SOD2*. (H) Expression of HIF-2α target genes *PAI1*, *CITED2* and *SOD2* in 786-O cells was not affected by bacteria-delivered NleB. 786-O cells were infected with the mutant EPEC strains Δ*nleBE*+vector or Δ*nleB*+pNleB, respectively. Δ*nleBE*+vector, a *nleE-* and *nleB-*deleted mutant EPEC strain (strain SC309, indicated as Δ*nleBE*) complemented with an empty plasmid; Δ*nleBE*+pNleB, Δ*nleBE* complemented with a plasmid expressing wild-type NleB. Data are presented as means + SEM of three independent experiments performed in triplicate.
(TIF)

S12 Fig PX-478 treatment causes a reduction of Hif-1α protein levels and decreases HIF-1α downstream gene expression, but does not affect colonization of *C*. *rodentium* in mouse colon. (A) Injection of the Hif-1α inhibitor PX-478 reduced Hif-1α protein levels in mouse colon. C57BL/6 mice (5–6 weeks old; 17–19 g/mouse) were injected intraperitoneally with PX-478 (30 μg/g) for 4 days (the same amount of injection, 2 day intervals). Protein levels of Hif-1α in mouse colon were detected by western blot. (B) Expressions of *pdk1*, *slc2a1*, and *pkm2* were significantly decreased in mouse colon after injection of PX-478 compared with the control, but expression of *sod2* was not altered. (C, D) After injection of PX-478 (the second time), the mice were orally gavaged with indicated wild-type *C*. *rodentium* strain. Viable stool bacterial counts measured at days 4, 8, 12, and 16 after inoculation are shown with mean ± SEM of log_10_ colony-forming units (CFU) per gram feces (C). Bacterial colonization in the intestine after infection for 8 days is shown as the mean ± SEM of log_10_ CFU per gram colon (n > 6) (D). P-values were determined by Student’s *t* test.
(TIF)

S13 Fig NleB enhances host glucose metabolism *in vivo*. (A) Male C57BL/6 mice (5–6 weeks old; 17–19 g/mouse) were orally gavaged with the wild-type *C*. *rodentium* DBS100 strain or Δ*nleB* strain; the mutant strains were complemented with a plasmid expressing wild-type NleB (pNleBc) or the GlcNAc transferase-deficient D221A/D223A mutant (pNleBc-DXD). Blood glucose levels were measured (before fasting). P-values were calculated by ANOVA (B) After fasting for 6 h, serum glucose levels were measured again (n = 7 mice for each group, data combined from two independent experiments). P-values were determined by ANOVA. (C) Male C57BL/6 mice (5–6 weeks old; 17–19 g/mouse) were injected intraperitoneally with PX-478 (30 μg/g) for 48 h, followed by oral gavage with the indicated *C*. *rodentium* strains. Serum glucose levels were measured (before fasting). (D) After fasting for 6 h, serum glucose levels were measured again (n = 7 mice for each group, data combined from two independent experiments). (E, F) After glucose measurement, mouse livers were dissected, and liver *slc2a1* (E) or *pkm2* (F) expression was examined by semi-quantitative real-time PCR assays. (G) Injection of the Hif-1α inhibitor PX-478 reduced Hif-1α protein levels in mouse liver. C57BL/6 mice (5–6 weeks old, 17–19 g/mouse) were injected intraperitoneally with PX-478 (30 μg/g). After completing blood glucose measurements, the mice were dissected and the livers were harvested to examine Hif-1α levels by western blot with an anti-Hif-1α antibody.
(TIF)

S14 Fig NleB does not affect β cell proliferation in the pancreas and does not alter serum insulin levels. (A) Pancreas samples from mice infected with the wild-type *C*. *rodentium* (WT), the nleB mutant (ΔnleB), or uninfected were stained with the proliferation marker KI67 (red) and insulin marker (green) to distinguish beta islets. Arrowheads denote proliferating β cells. (B) Insulin was measured in blood of mice after infection with *C*. *rodentium* WT, ΔnleB, or uninfected for 2 or 4 days (n = 5 each group). (C) Homeostasis model assessment of insulin resistance (HOMA-IR) levels were calculated for mice after infection with *C*. *rodentium* WT or ΔnleB (n = 5 each group). Error bars indicate SEM.
(TIF)
