# Supplementary material for: A pathogen-derived effector modulates host glucose metabolism by arginine GlcNAcylation of HIF-1α protein
Source: PLoS Pathog. 2018 Aug 20;14(8):e1007259. doi: 10.1371/journal.ppat.1007259 (PMC6117090; doi:10.1371/journal.ppat.1007259)
Supplement: S1 Table — (DOC) [file ppat.1007259.s001.doc]

**S1 Table. Bacterial strains used in this study**

| **Strain characteristics source** |
| --- |

***Escherichia coli***

**EPEC E2348/69 Wild-type EPEC::Nal^r^ (1)**

**△nleBE EPEC E2348/69 △nleBE::Kan^r^,Tet^r^** **(1)**

**△escN+pNleB EPEC E2348/69 △escN+pNleB:: kan^r^ ,Amp^r^** **(1)**

**△nleBE+pNleB EPEC E2348/69 △nleBE+pNleB::Kan^r^ ,Tet^r^,Amp^r^ (1)**

**△nleBE+pNleB-DXD EPEC E2348/69 △nleBE+pNleB-DXD::Kan^r^,Tet^r^,Amp^r^ (1)**

**△nleBE+HA-pNleB EPEC E2348/69 △nleBE+HA-pNleB::Kan^r^,Tet^r^,gen^r^ this study**

**△nleBE+HA-pNleB-DXD EPEC E2348/69 △nleBE+HA-pNleB-DXD::Kan^r^,Tet^r^,gen^r^ this study**

***Citrobacter rodentium***

**DBS100 Wild-tyeCitrobacterrodentium(ATCC51459)::Nal^r^,Cm^r^ (1)**

**△nleB DBS100 △nleB::Nal^r^ ,Cm^r^ (1)**

**△nleB+pNleBc DBS100 △nleB+pNleBc::Nal^r^ ,Cm^r^,Kan^r^ (1)**

**△nleB+pNleBc-DXD DBS100 △nleB+pNleBc-DXD::Nal^r^ ,Cm^r^,Kan^r^ (1)**

**△nleB+HA-pNleBc DBS100 △nleB+HA-pNleBc-DXD::Nal^r^ ,Cm^r^,gen^r^ this study**

**△nleB+HA-pNleBc-DXD DBS100 △nleBc+HA-pNleBc-DXD::Nal^r^ ,Cm^r^,gen^r^ this study**

1. **S. Li, L. Zhang, Q. Yao, L. Li, N. Dong, J. Rong, W. Gao, X.Ding, L. Sun, X. Chen, F. Shao, Nature.501,242–246(2013)**
