# Supplementary material for: A pathogen-derived effector modulates host glucose metabolism by arginine GlcNAcylation of HIF-1α protein
Source: PLoS Pathog. 2018 Aug 20;14(8):e1007259. doi: 10.1371/journal.ppat.1007259 (PMC6117090; doi:10.1371/journal.ppat.1007259)
Supplement: S2 Table — (DOC) [file ppat.1007259.s002.doc]

**S2 Table. Summary of observed modification in peptides of HIF-1α purified from immuno-precipitation of HEK293T cells by MS/MS analysis. Charge, spectra mass, theory mass, delta mass and PEP-score were presented.**

**
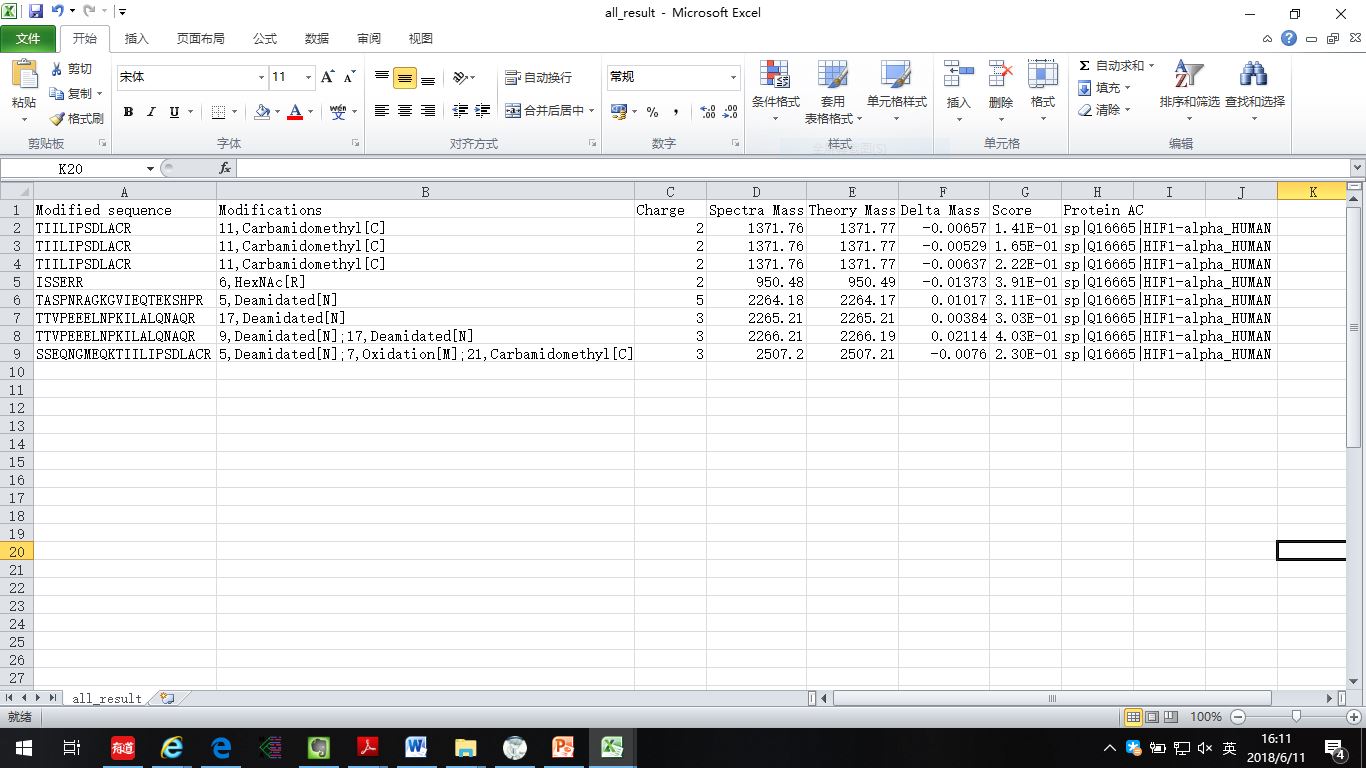
**
