# Supplementary figures and images for: A pathogen-derived effector modulates host glucose metabolism by arginine GlcNAcylation of HIF-1α protein
Source: PLoS Pathog. 2018 Aug 20;14(8):e1007259. doi: 10.1371/journal.ppat.1007259 (PMC6117090; doi:10.1371/journal.ppat.1007259)

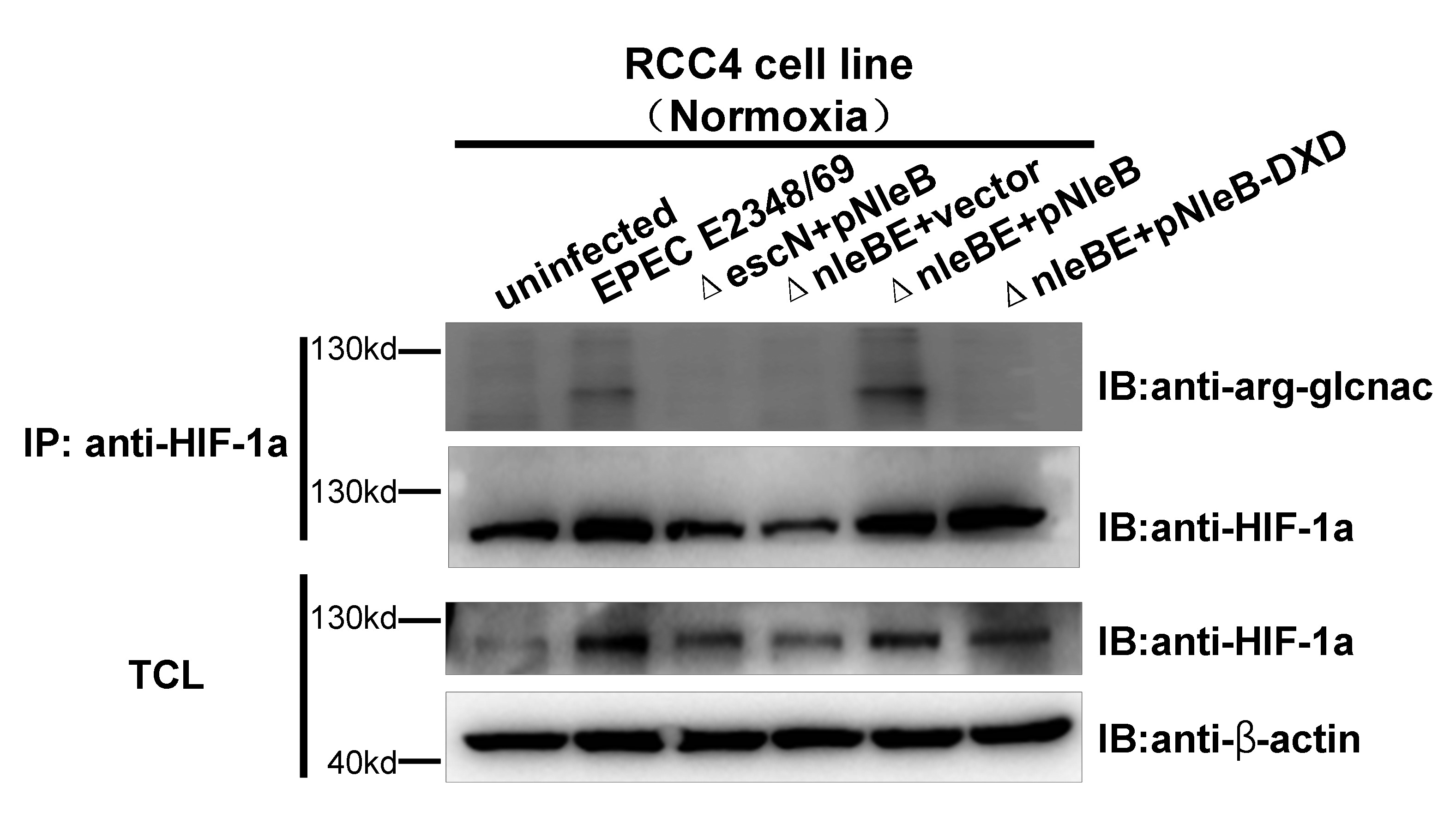

Supplement: S1 Fig — RCC4 cells were uninfected or infected with the wild-type EPEC strain (EPEC E2348/69); a mutant EPEC strain lacking escN (indicated as ΔescN) but complemented with a plasmid expressing WT NleB (ΔescN + pNleB); or a mutant EPEC strain lacking both nleE and nleB (strain SC309, indicated as ΔnleBE) but complemented with an empty plasmid (ΔnleBE + vector), a plasmid expressing wild-type NleB (ΔnleBE+pNleB), ora plasmid expressing the GlcNAc transferase-deficient D221A/D223A mutant (ΔnleBE+pNleB-DXD). The cell lysates were subjected to anti-HIF-1α IP and detected by an anti-arginine (GlcNAc) antibody. IP, immunoprecipitation; TCL, total cell lysates; IB, immunoblotting. (TIF) [file ppat.1007259.s003.tif]

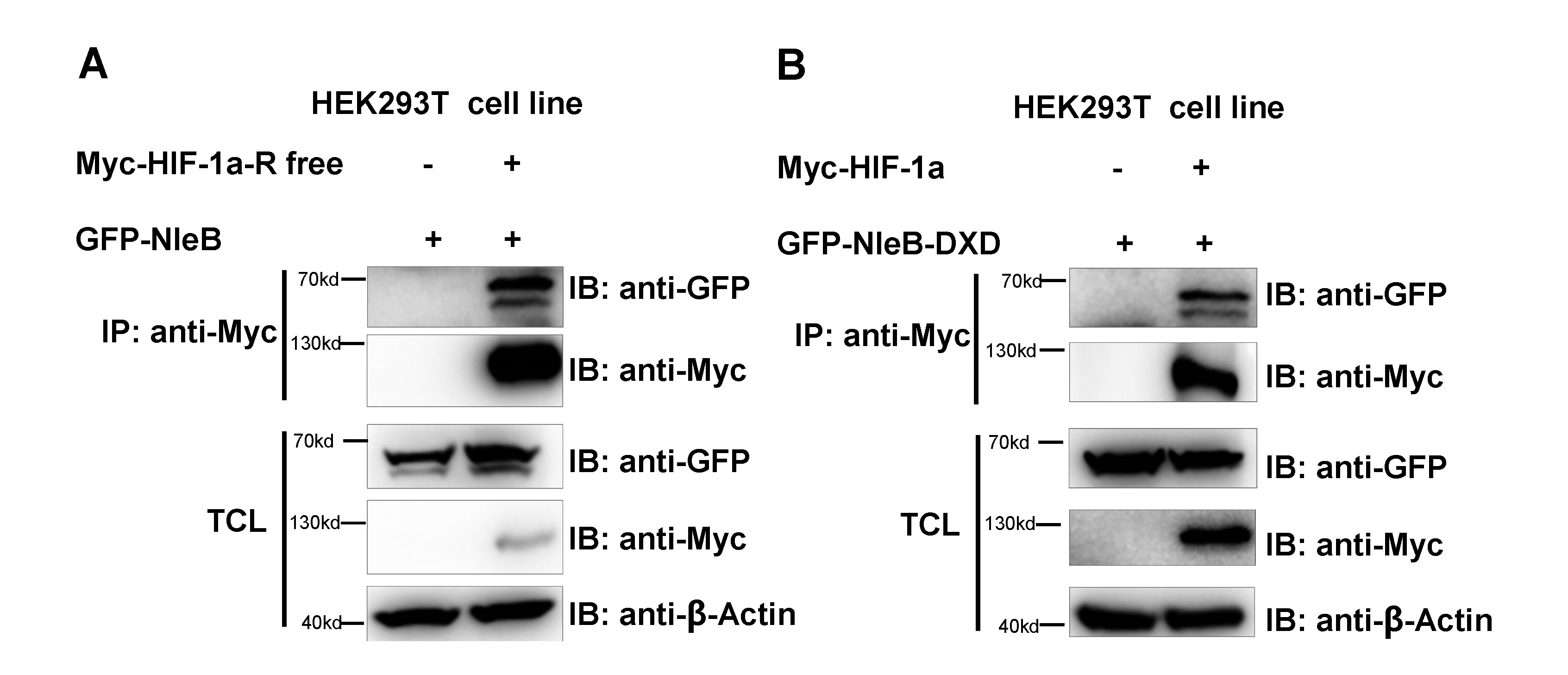

Supplement: S2 Fig — (A) NleB interacted with the HIF-1α-R-free mutant. (B) The GlcNAc transferase-deficient NleB, NleB-DXD, interacted with wild-type HIF-1α. HEK293T cells were transfected with the indicated plasmids. Anti-Myc antibody-conjugated agarose beads were used for co-IP, and anti-GFP antibody was used for detection. In HIF-1α-R-free, all 35 arginine residues in human HIF-1α were simultaneously mutated to lysine residues; NleB-DXD, NleB Asp221Ala/Asp223Ala double mutant; IP, immunoprecipitation; TCL, total cell lysates; GFP-NleB, GFP-tagged wild-type NleB. (TIF) [file ppat.1007259.s004.tif]

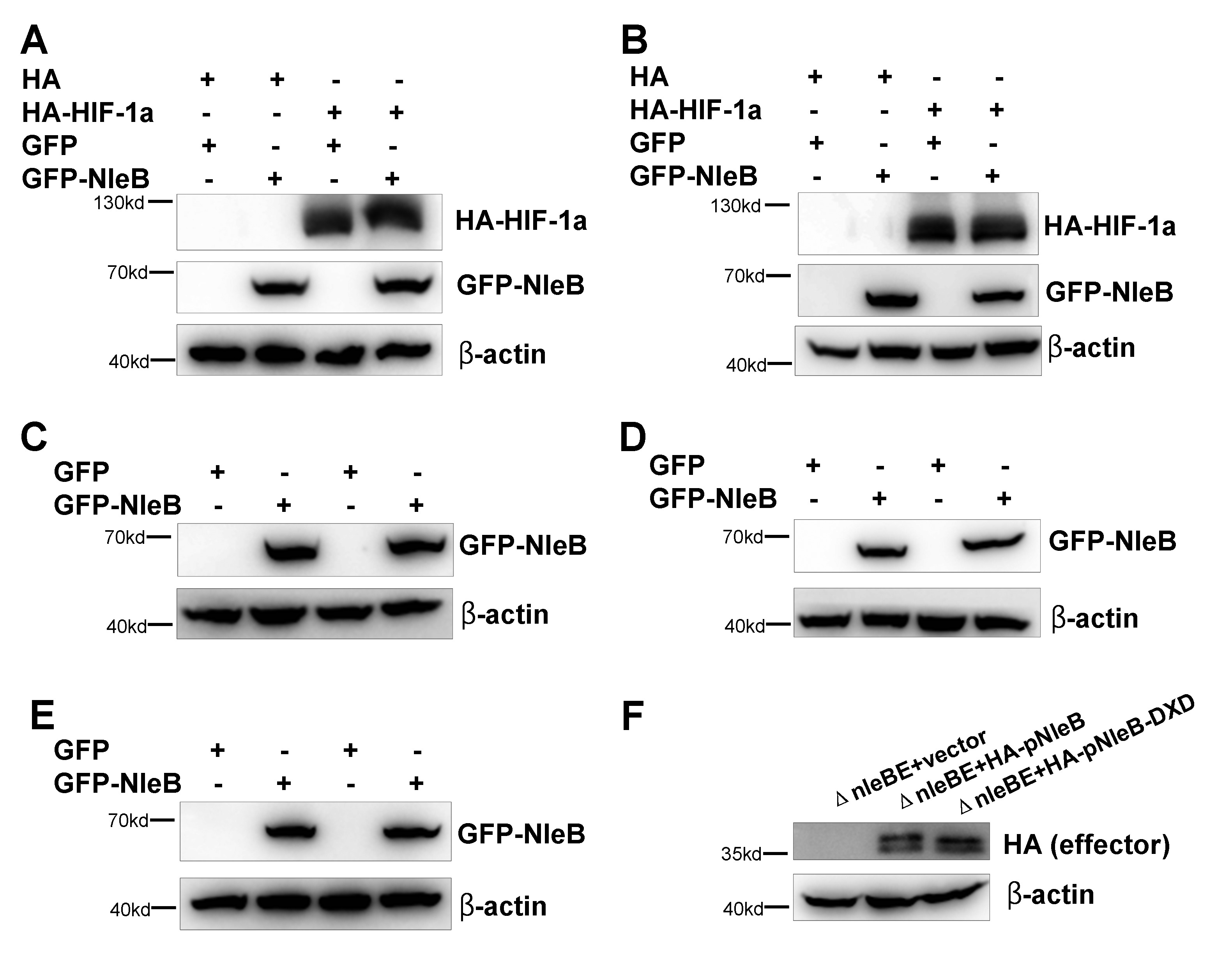

Supplement: S3 Fig — (A) Expressions of HIF-1α and NleB were confirmed in HCT116 cells after transfection for promoter assays (Fig 4A). (B) Expressions of HIF-1α and NleB were confirmed in HCT116 cells after transfection for promoter assays (Fig 4B). (C) Expression of NleB was confirmed in HCT116 cells after transfection for promoter assays (Fig 4C). (D) Expression of NleB was confirmed in HCT116 cells after transfection for promoter assays (Fig 4D). (E)Expression of NleB was confirmed in HCT116 cells after transfection for RT-PCR assays (Fig 4E–4H). (F) The levels of NleB and NleB-DXD in HCT116 cells translocated from the EPEC mutant ΔnleBE+HA-pNleB and the EPEC mutant ΔnleBE+HA-pNleB-DXD respectively as revealed by western blot assays using anti-HA antibody. (TIF) [file ppat.1007259.s005.tif]

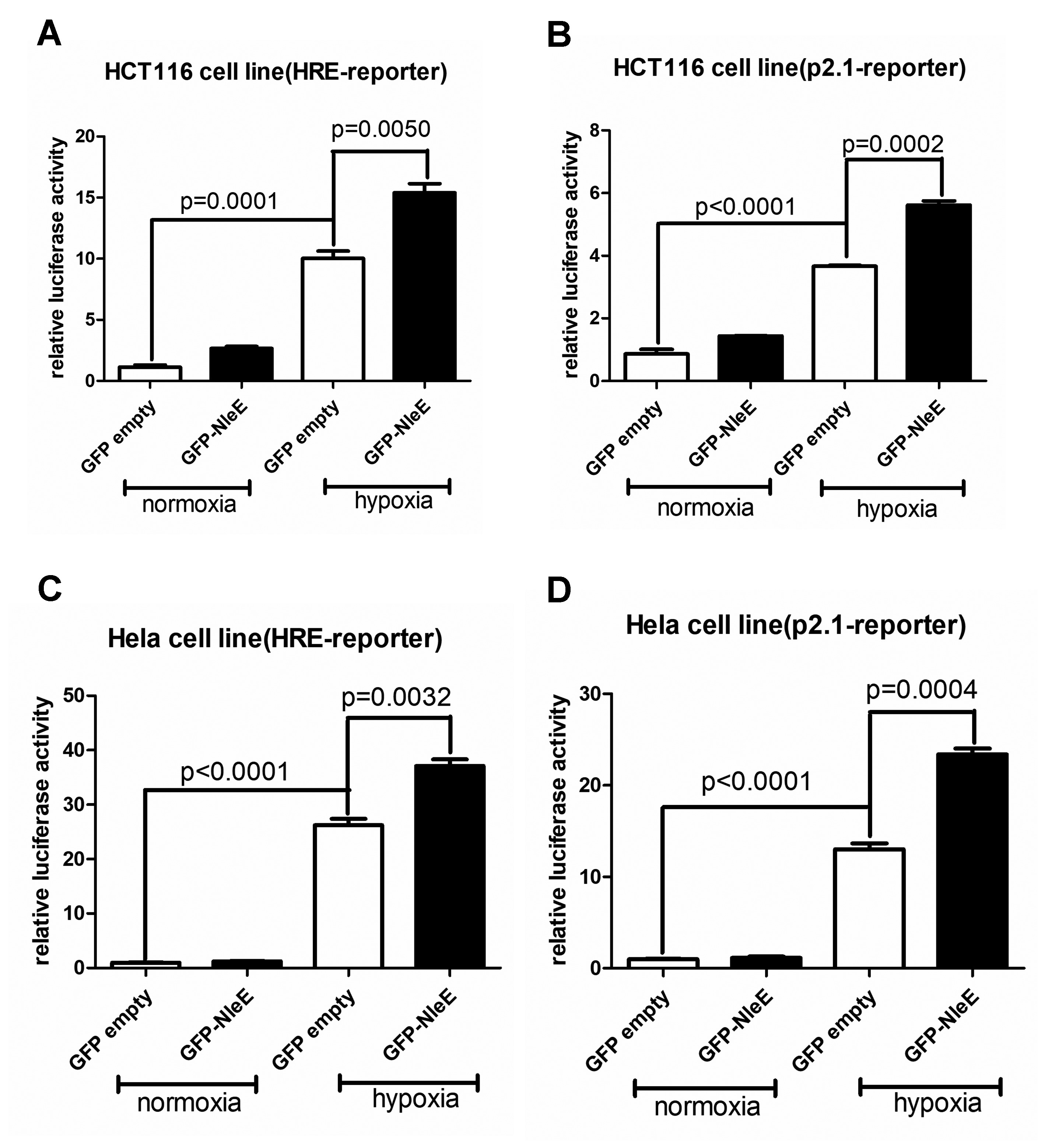

Supplement: S4 Fig — (A, B) Induction of HRE-reporter luciferase activity (A) or p2.1-reporter luciferase activity (B) under hypoxia was significantly enhanced by NleB transfection in HCT116 cells (p = 0.0050 and p = 0.0002, respectively). (C, D) Induction of HRE-reporter luciferase activity (C) or p2.1-reporter luciferase activity (D) under hypoxia was significantly enhanced by NleB transfection in HeLa cells (p = 0.0032 and p = 0.0004, respectively). (TIF) [file ppat.1007259.s006.tif]

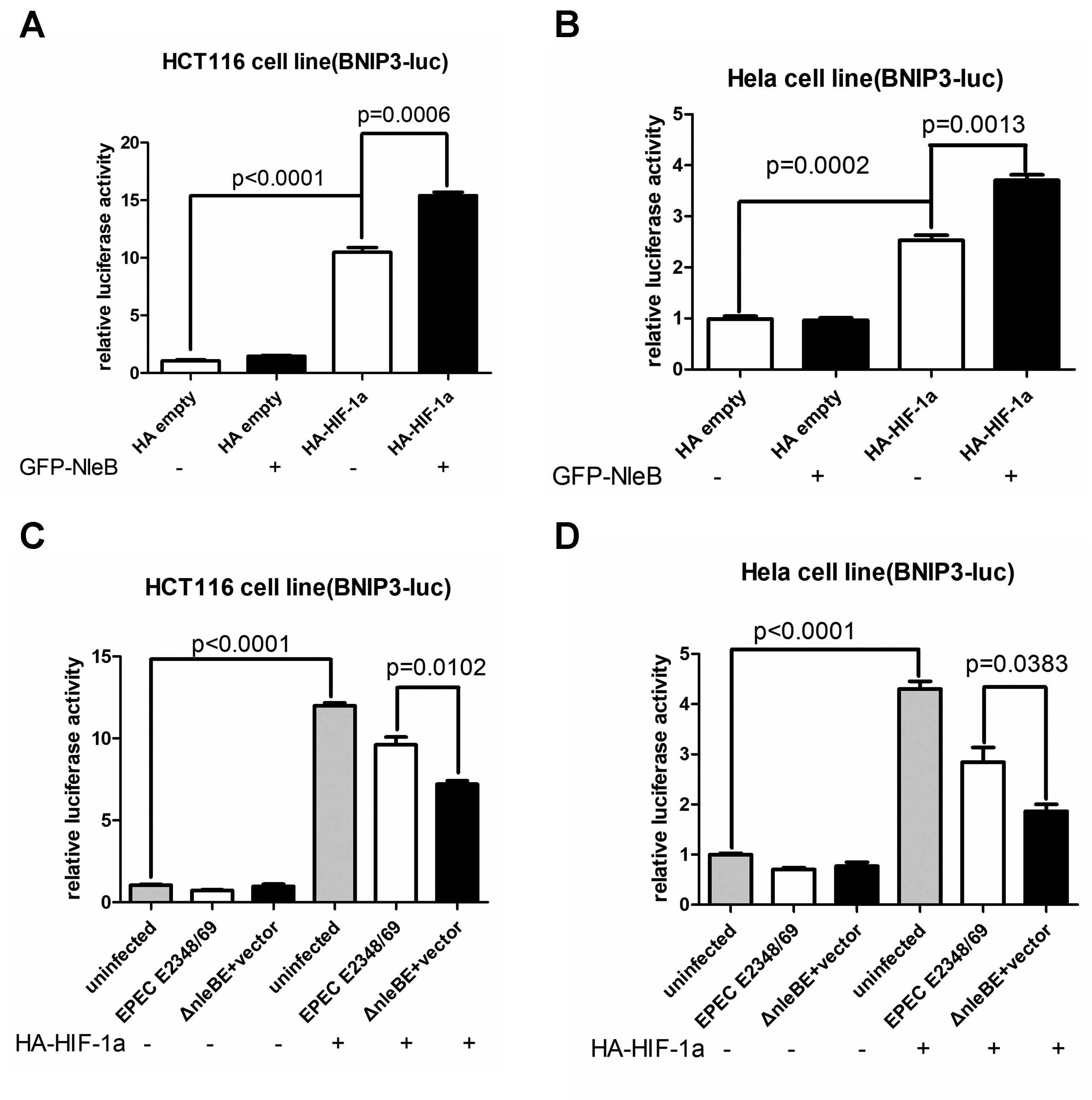

Supplement: S5 Fig — (A, B) Induction of BNIP-reporter luciferase activity by HIF-1α transfection was significantly enhanced by NleB transfection in HCT116 cells or HeLa cells (p = 0.0006 and p = 0.0013, respectively). (C, D) Induction of BNIP-reporter luciferase activity by HIF-1α transfection was significantly enhanced by infection with the wild-type EPEC strain (EPEC E2348/69) compared to infection with the mutant EPEC strain lacking both nleE and nleB (strain SC309) but complemented with an empty plasmid (ΔnleBE+vector) in HCT116 cells or HeLa cells (p = 0.0102 and p = 0.0383, respectively). (TIF) [file ppat.1007259.s007.tif]

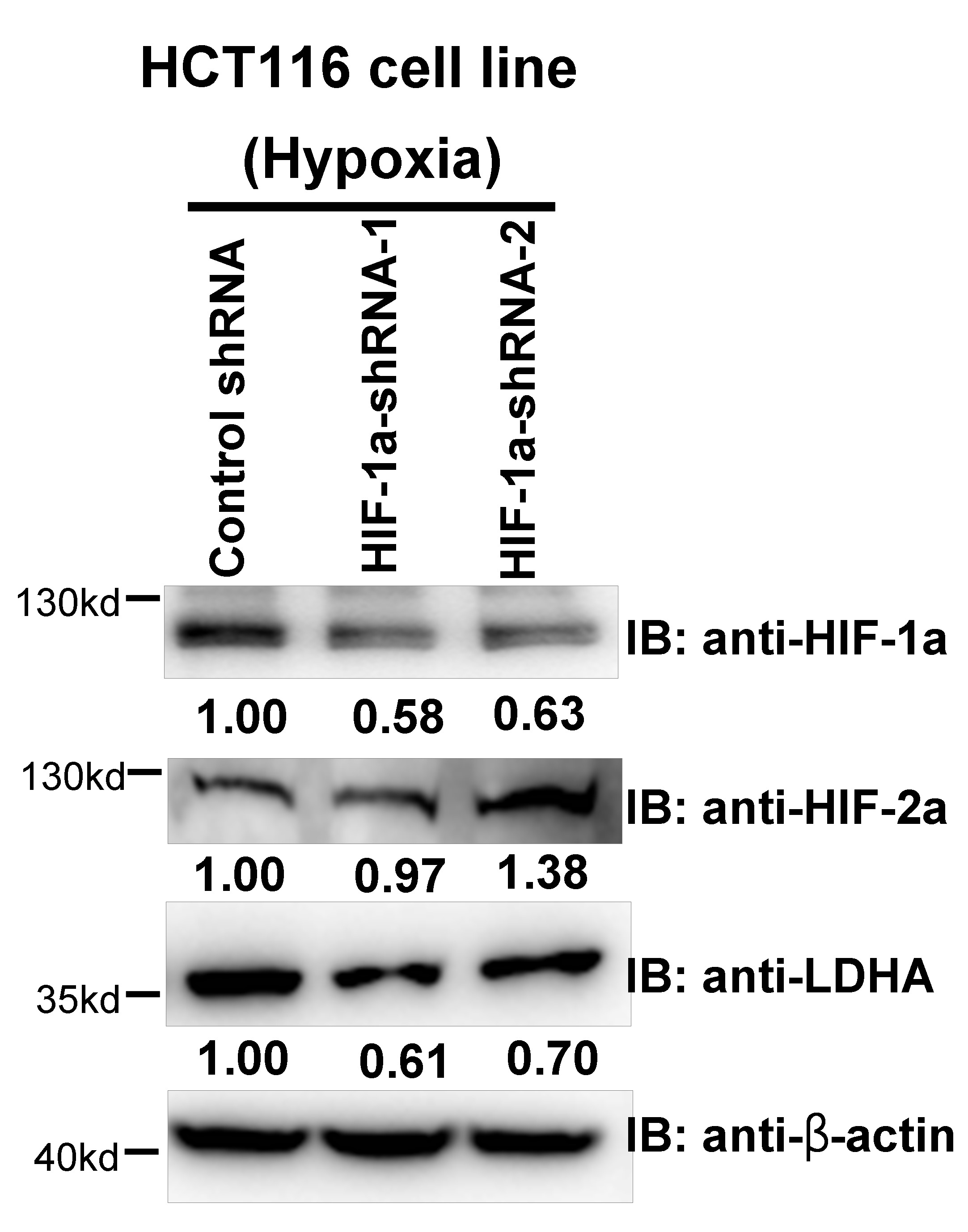

Supplement: S6 Fig — (TIF) [file ppat.1007259.s008.tif]

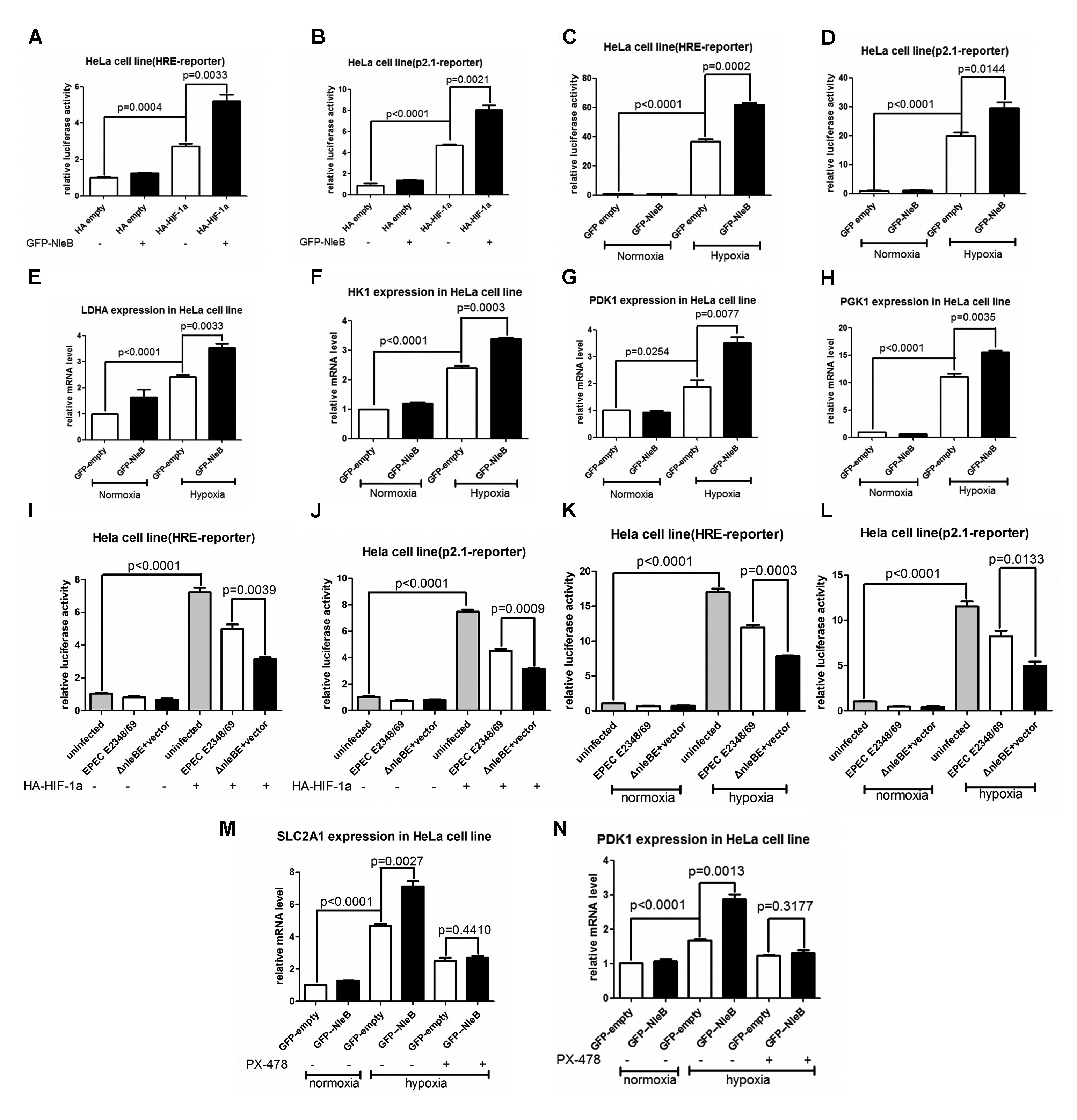

Supplement: S7 Fig — (A, B) Induction of HRE-reporter luciferase activity (A) or p2.1-reporter luciferase activity (B) by HIF-1α transfection under normoxia was significantly enhanced by NleB transfection in HeLa cells (p = 0.0033 and p = 0.0021, respectively). HRE, hypoxia response element. (C, D) Induction of HRE-reporter luciferase activity (C) or p2.1-reporter luciferase activity (D) under hypoxia was significantly enhanced by NleB transfection in HeLa cells (p = 0.0002 and p = 0.0144, respectively). (E, F, G, H) Induction of LDHA (E), HK1(F), PDK1 (G), or PGK1 (H) mRNA expression under hypoxia was significantly enhanced by NleB transfection in HeLa cells (p = 0.0033, 0.0003, 0.0077, and 0.0035, respectively). (I, J) Induction of HRE-reporter luciferase activity (I) or p2.1-reporter luciferase activity (J) by HIF-1α transfection under normoxia in HeLa cells was significantly enhanced by infection with the wild-type EPEC strain (EPEC E2348/69) compared to infection with the mutant EPEC strain lacking both nleE and nleB (strain SC309) but complemented with an empty plasmid (ΔnleBE+vector) (p = 0.0039 and p = 0.0009, respectively). (K, L) Induction of HRE-reporter luciferase activity (K) or p2.1-reporter luciferase activity (L) in HeLa cells infected with EPEC E2348/69 was significantly enhanced under hypoxia (p<0.0003 and p = 0.0133, respectively). (M, N) The HIF-1α inhibitor PX-478 (25μM) blocked the enhancement of SLC2A1 (M) or PDK1 (N) mRNA expression by NleB transfection under hypoxia in HeLa cells (p = 0.4410 and p = 0.3177, respectively). Data are presented as means + SEM of three independent experiments performed in triplicate. (TIF) [file ppat.1007259.s009.tif]

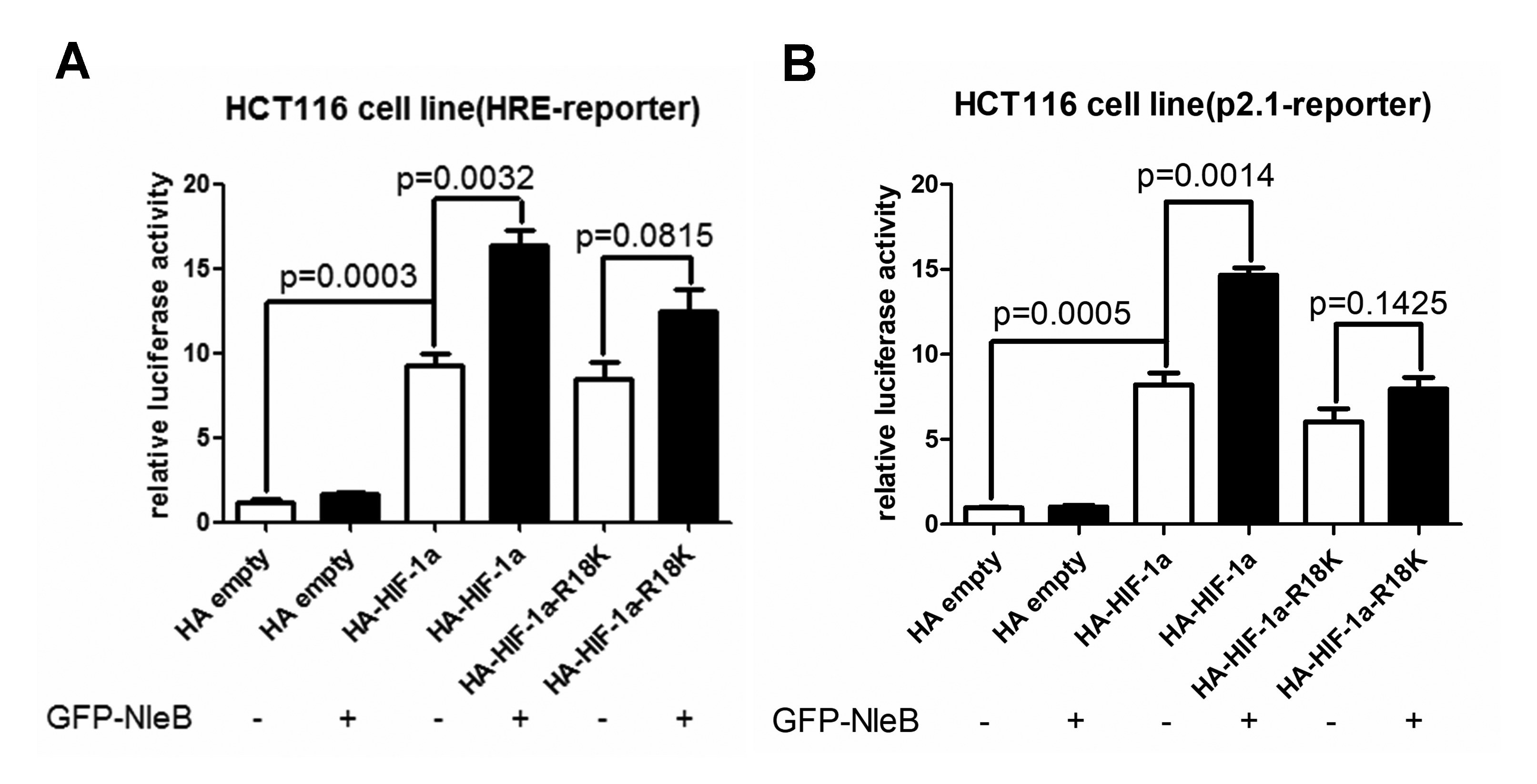

Supplement: S8 Fig — (A) Induction of HRE-reporter luciferase activity by HIF-1α (R18K) mutant transfection under normoxia was not significantly enhanced by NleB transfection in HCT116 cells (p = 0.0815) compared with that by wild-type HIF-1α (p = 0.0032). HRE, hypoxia response element. (B) Induction of p2.1-reporter luciferase activity by HIF-1α (R18K) mutant transfection under normoxia was not significantly enhanced by NleB transfection in HCT116 cells (p = 0.1425) compared with that by wild-type HIF-1α (p = 0.0014). Data are presented as means + SEM of three independent experiments performed in triplicate. (TIF) [file ppat.1007259.s010.tif]

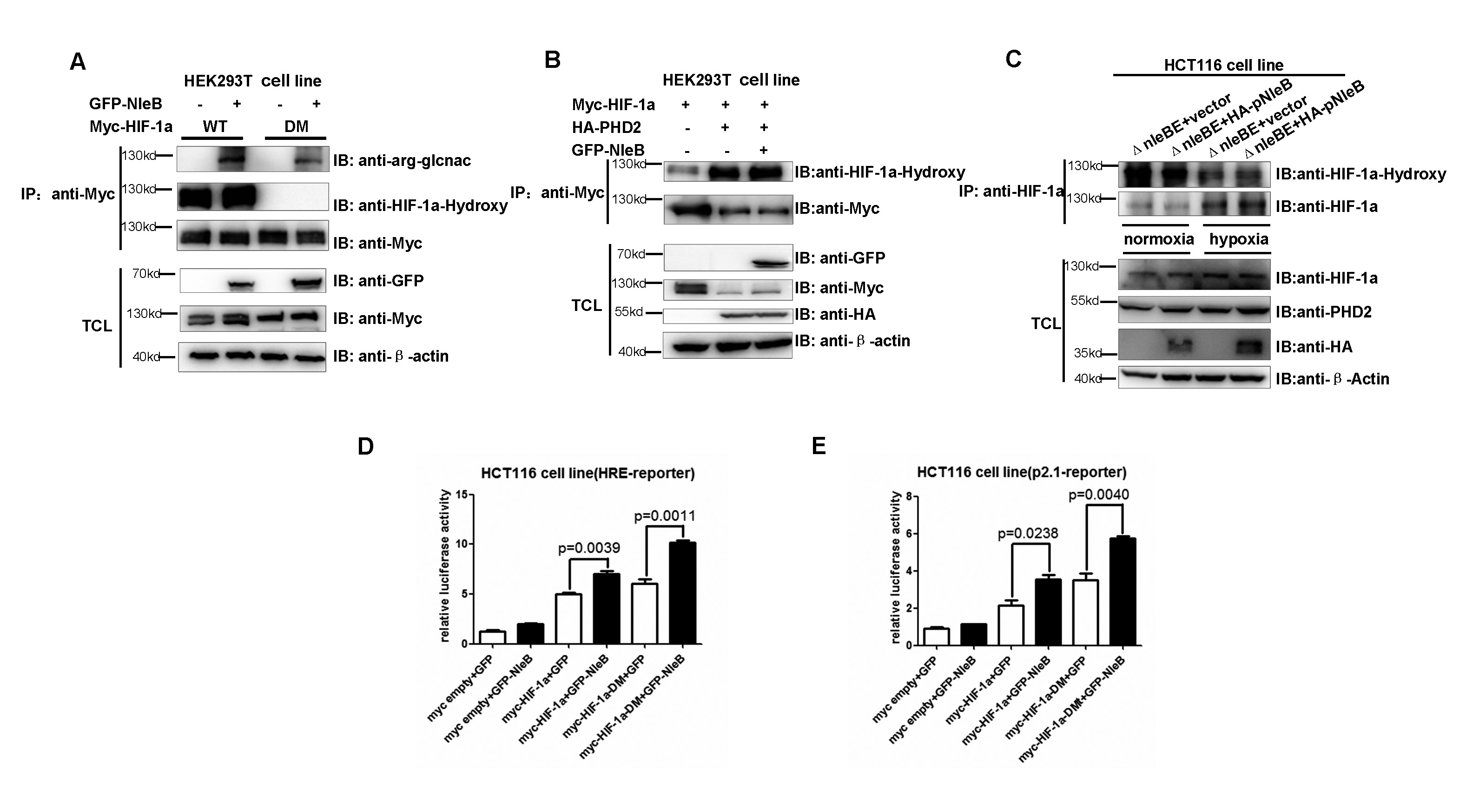

Supplement: S9 Fig — (A) Effects of NleB transfection on arginine GlcNAcylation of the hydroxylated site-mutated HIF-1α (DM) in HEK293T cells. IP, immunoprecipitation; TCL, total cell lysates; GFP-NleB, GFP-tagged wild-type NleB; WT, wild-type HIF-1α; DM, a HIF-1α mutant with two proline residues mutated to alanine residues (P402A/P564A). (B) Effects of NleB transfection on hydroxylation of HIF-1α by PHD2 in HEK293T cells. (C) Effects of NleB on hydroxylation of endogenous HIF-1α in HCT116 cells after infection with the indicated EPEC strains under either normoxia or hypoxia. (D) Induction of HRE-reporter luciferase activity by HIF-1α-DM transfection under normoxia was significantly enhanced by NleB transfection in HCT116 cells (p = 0.0011). HRE, hypoxia response element. (E) Induction of p2.1-reporter luciferase activity by HIF-1α-DM transfection under normoxia was significantly enhanced by NleB transfection in HCT116 cells (p = 0.0040). Data are presented as means + SEM of three independent experiments performed in triplicate. (TIF) [file ppat.1007259.s011.tif]

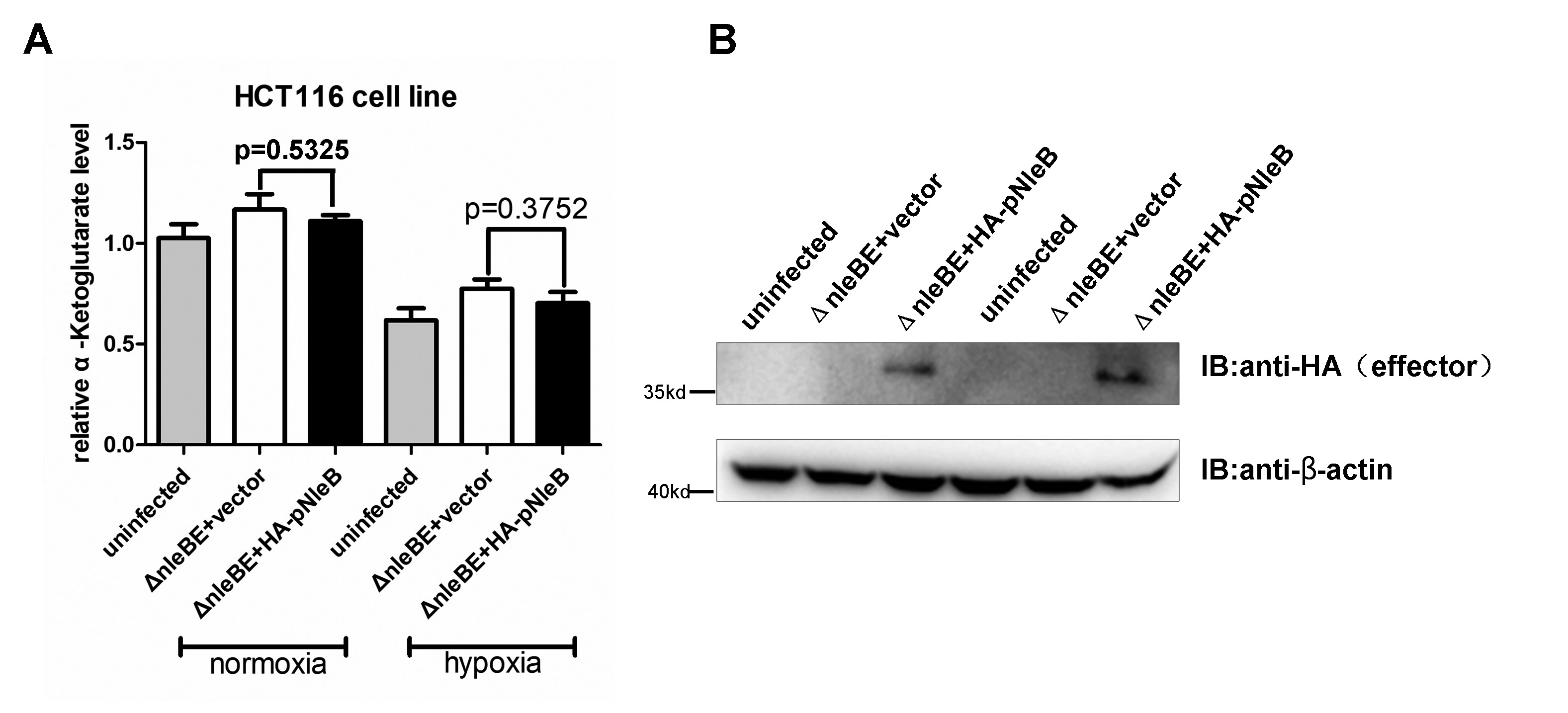

Supplement: S10 Fig — (A) Cellular α-ketoglutarate levels in HCT116 cells were measured after infection or without infection with mutant EPEC strains lacking both nleE and nleB (strain SC309, indicated as ΔnleBE) but complemented with an empty plasmid (ΔnleBE+vector) or a plasmid expressing wild-type HA-tagged NleB (ΔnleBE+HA-pNleB) for 4 h under normoxia or hypoxia (the cells were pre-treated under hypoxia for 12 h before infection). (B) Expression of HA-NleB was confirmed by western blot. (TIF) [file ppat.1007259.s012.tif]

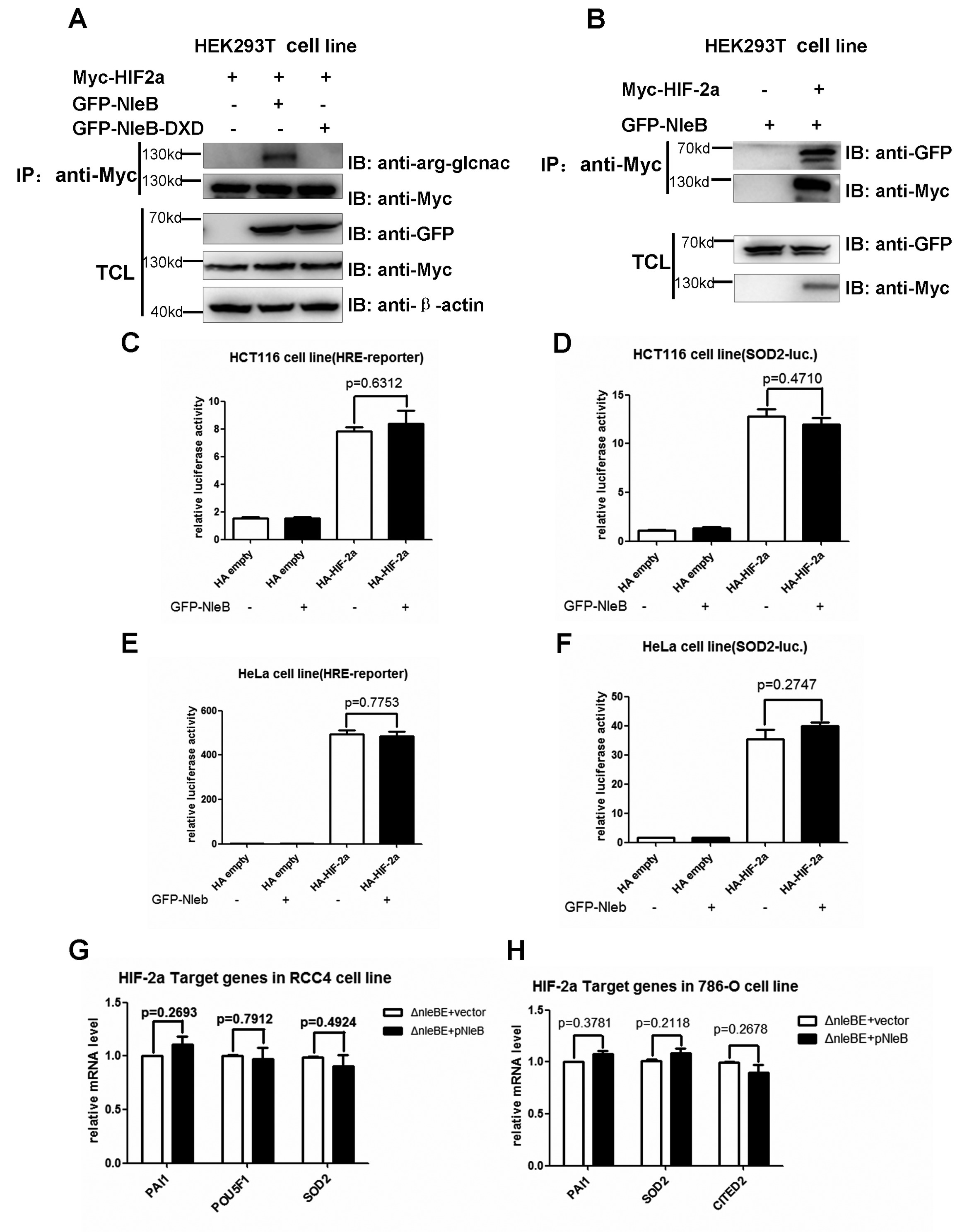

Supplement: S11 Fig — (A) Effects of NleB transfection on arginine GlcNAcylation of HIF-2α in HEK293T cells. IP, immunoprecipitation; TCL, total cell lysates; GFP-NleB, GFP-tagged wild-type NleB; GFP-BleB-DXD, GFP-tagged NleB Asp221Ala/Asp223Ala double mutant. (B) NleB interacted with HIF-2α. HEK293T cells were transfected with the indicated plasmids. Anti-Myc antibody-conjugated agarose beads were used for co-immunoprecipitation, and anti-GFP and anti-Myc antibodies were used for detection. (C) Induction of HRE-reporter luciferase activity by HIF-2α transfection under normoxia was not affected by NleB transfection in HCT116 cells (p = 0.6312). HRE, hypoxia response element. (D) Induction of SOD2-reporter luciferase activity by HIF-2α transfection under normoxia was not affected by NleB transfection in HCT116 cells (p = 0.4710). (E) Induction of HRE-reporter luciferase activity by HIF-2α transfection under normoxia was not affected by NleB transfection in HeLa cells (p = 0.7753). (F) Induction of SOD2-reporter luciferase activity by HIF-2α transfection under normoxia was not affected by NleB transfection in HeLa cells (p = 0.2747). (G) Expression of HIF-2α target genes PAI1, POU5F1, and SOD2 in RCC4 cells was not affected by bacteria-delivered NleB. RCC4 cells were infected with the mutant EPEC strains ΔnleBE+vector or ΔnleB+pNleB, respectively; semi-quantitative RT-PCR was used to detect mRNA levels of PAI1, POU5F1, and SOD2. (H) Expression of HIF-2α target genes PAI1, CITED2 and SOD2 in 786-O cells was not affected by bacteria-delivered NleB. 786-O cells were infected with the mutant EPEC strains ΔnleBE+vector or ΔnleB+pNleB, respectively. ΔnleBE+vector, a nleE- and nleB-deleted mutant EPEC strain (strain SC309, indicated as ΔnleBE) complemented with an empty plasmid; ΔnleBE+pNleB, ΔnleBE complemented with a plasmid expressing wild-type NleB. Data are presented as means + SEM of three independent experiments performed in triplicate. (TIF) [file ppat.1007259.s013.tif]

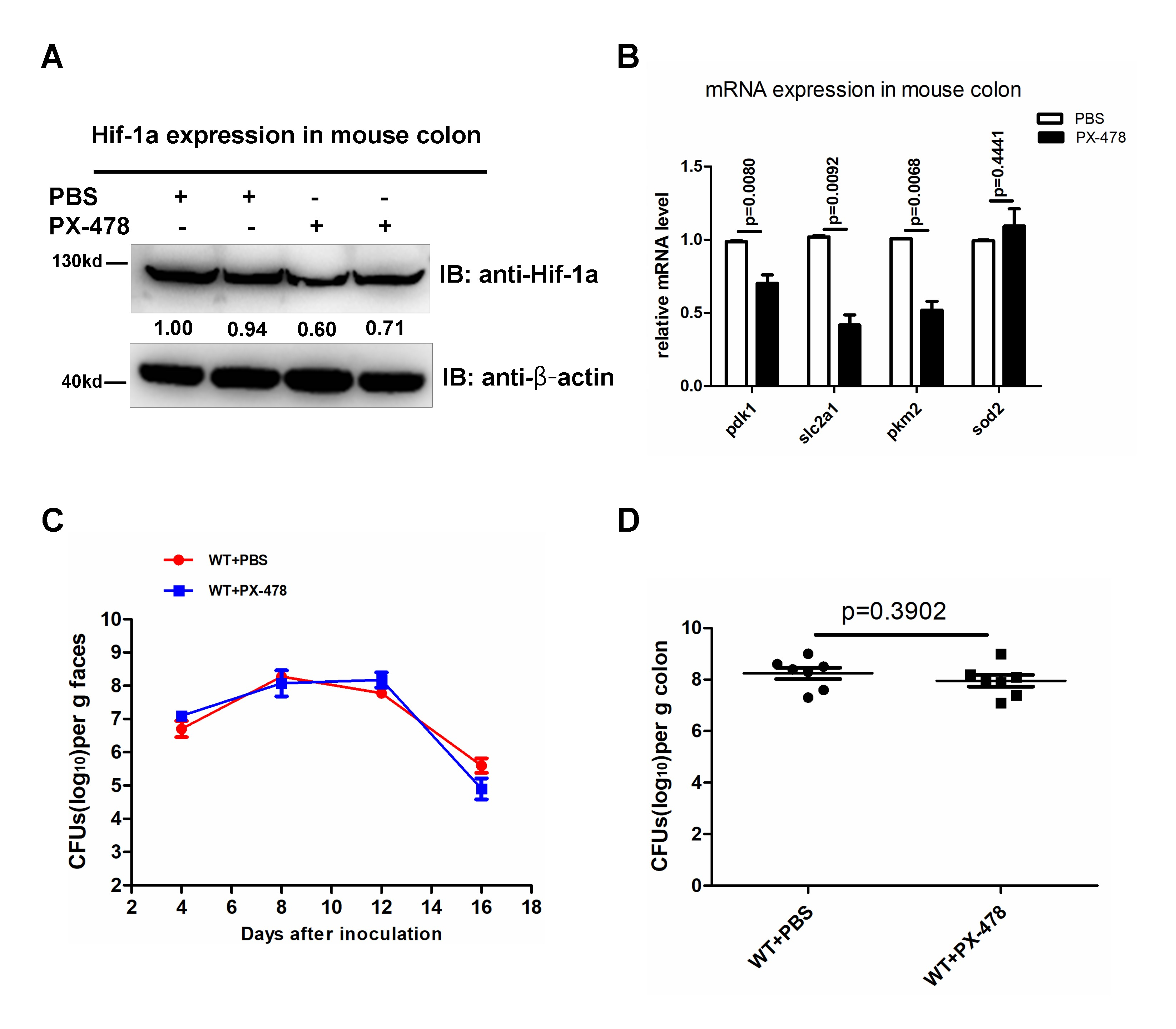

Supplement: S12 Fig — (A) Injection of the Hif-1α inhibitor PX-478 reduced Hif-1α protein levels in mouse colon. C57BL/6 mice (5–6 weeks old; 17–19 g/mouse) were injected intraperitoneally with PX-478 (30 μg/g) for 4 days (the same amount of injection, 2 day intervals). Protein levels of Hif-1α in mouse colon were detected by western blot. (B) Expressions of pdk1, slc2a1, and pkm2 were significantly decreased in mouse colon after injection of PX-478 compared with the control, but expression of sod2 was not altered. (C, D) After injection of PX-478 (the second time), the mice were orally gavaged with indicated wild-type C. rodentium strain. Viable stool bacterial counts measured at days 4, 8, 12, and 16 after inoculation are shown with mean ± SEM of log10 colony-forming units (CFU) per gram feces (C). Bacterial colonization in the intestine after infection for 8 days is shown as the mean ± SEM of log10 CFU per gram colon (n > 6) (D). P-values were determined by Student’s t test. (TIF) [file ppat.1007259.s014.tif]

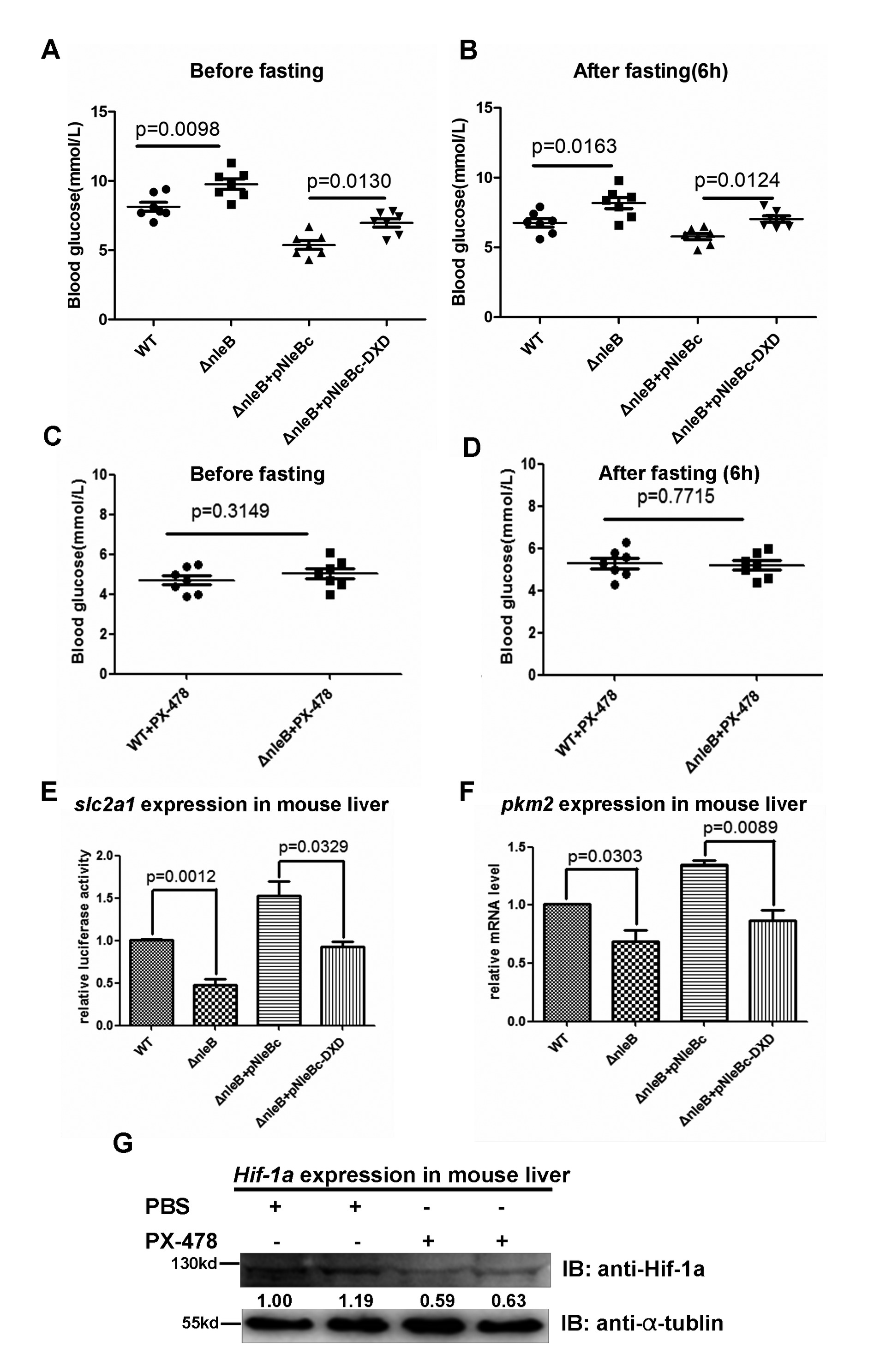

Supplement: S13 Fig — (A) Male C57BL/6 mice (5–6 weeks old; 17–19 g/mouse) were orally gavaged with the wild-type C. rodentium DBS100 strain or ΔnleB strain; the mutant strains were complemented with a plasmid expressing wild-type NleB (pNleBc) or the GlcNAc transferase-deficient D221A/D223A mutant (pNleBc-DXD). Blood glucose levels were measured (before fasting). P-values were calculated by ANOVA (B) After fasting for 6 h, serum glucose levels were measured again (n = 7 mice for each group, data combined from two independent experiments). P-values were determined by ANOVA. (C) Male C57BL/6 mice (5–6 weeks old; 17–19 g/mouse) were injected intraperitoneally with PX-478 (30 μg/g) for 48 h, followed by oral gavage with the indicated C. rodentium strains. Serum glucose levels were measured (before fasting). (D) After fasting for 6 h, serum glucose levels were measured again (n = 7 mice for each group, data combined from two independent experiments). (E, F) After glucose measurement, mouse livers were dissected, and liver slc2a1 (E) or pkm2 (F) expression was examined by semi-quantitative real-time PCR assays. (G) Injection of the Hif-1α inhibitor PX-478 reduced Hif-1α protein levels in mouse liver. C57BL/6 mice (5–6 weeks old, 17–19 g/mouse) were injected intraperitoneally with PX-478 (30 μg/g). After completing blood glucose measurements, the mice were dissected and the livers were harvested to examine Hif-1α levels by western blot with an anti-Hif-1α antibody. (TIF) [file ppat.1007259.s015.tif]

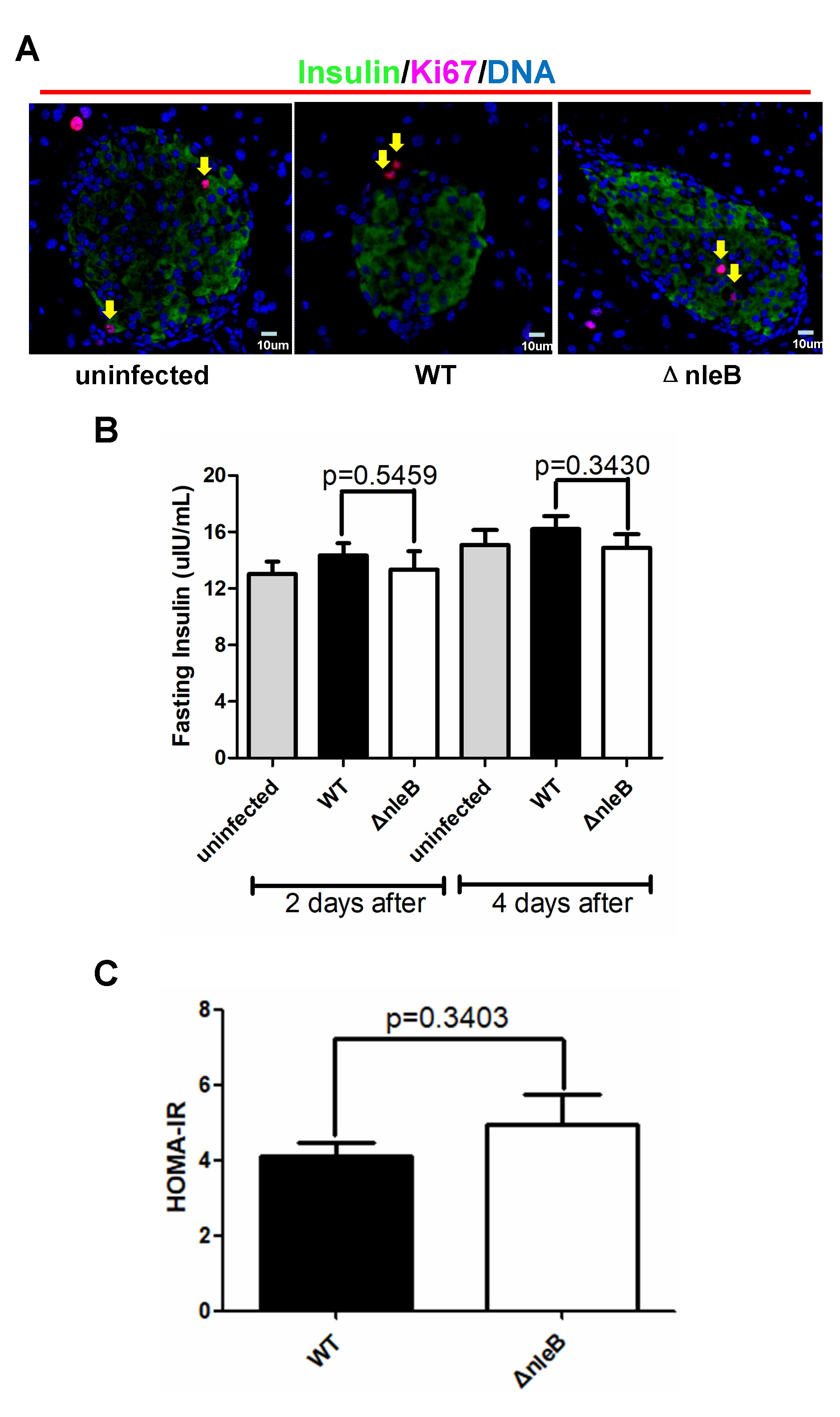

Supplement: S14 Fig — (A) Pancreas samples from mice infected with the wild-type C. rodentium (WT), the nleB mutant (ΔnleB), or uninfected were stained with the proliferation marker KI67 (red) and insulin marker (green) to distinguish beta islets. Arrowheads denote proliferating β cells. (B) Insulin was measured in blood of mice after infection with C. rodentium WT, ΔnleB, or uninfected for 2 or 4 days (n = 5 each group). (C) Homeostasis model assessment of insulin resistance (HOMA-IR) levels were calculated for mice after infection with C. rodentium WT or ΔnleB (n = 5 each group). Error bars indicate SEM. (TIF) [file ppat.1007259.s016.tif]
